# Detailed Evaluation of Data Analysis Tools for Subtyping of Bacterial Isolates Based on Whole Genome Sequencing: *Neisseria meningitidis* as a Proof of Concept

**DOI:** 10.3389/fmicb.2019.02897

**Published:** 2019-12-18

**Authors:** Assia Saltykova, Wesley Mattheus, Sophie Bertrand, Nancy H. C. Roosens, Kathleen Marchal, Sigrid C. J. De Keersmaecker

**Affiliations:** ^1^Transversal Activities in Applied Genomics, Sciensano, Brussels, Belgium; ^2^IDLab, IMEC, Department of Information Technology, Ghent University, Ghent, Belgium; ^3^Belgian National Reference Centre for Neisseria, Human Bacterial Diseases, Sciensano, Brussels, Belgium; ^4^Department of Plant Biotechnology and Bioinformatics, VIB, Ghent University, Ghent, Belgium

**Keywords:** *Neisseria meningitidis*, whole genome sequencing, public health, subtyping, data analysis, benchmarking, cgMLST, SNP

## Abstract

Whole genome sequencing is increasingly recognized as the most informative approach for characterization of bacterial isolates. Success of the routine use of this technology in public health laboratories depends on the availability of well-characterized and verified data analysis methods. However, multiple subtyping workflows are now often being used for a single organism, and differences between them are not always well described. Moreover, methodologies for comparison of subtyping workflows, and assessment of their performance are only beginning to emerge. Current work focuses on the detailed comparison of WGS-based subtyping workflows and evaluation of their suitability for the organism and the research context in question. We evaluated the performance of pipelines used for subtyping of *Neisseria meningitidis*, including the currently widely applied cgMLST approach and different SNP-based methods. In addition, the impact of the use of different tools for detection and filtering of recombinant regions and of different reference genomes were tested. Our benchmarking analysis included both assessment of technical performance of the pipelines and functional comparison of the generated genetic distance matrices and phylogenetic trees. It was carried out using replicate sequencing datasets of high- and low-coverage, consisting mainly of isolates belonging to the clonal complex 269. We demonstrated that cgMLST and some of the SNP-based subtyping workflows showed very good performance characteristics and highly similar genetic distance matrices and phylogenetic trees with isolates belonging to the same clonal complex. However, only two of the tested workflows demonstrated reproducible results for a group of more closely related isolates. Additionally, results of the SNP-based subtyping workflows were to some level dependent on the reference genome used. Interestingly, the use of recombination-filtering software generally reduced the similarity between the gene-by-gene and SNP-based methodologies for subtyping of *N. meningitidis*. Our study, where *N. meningitidis* was taken as an example, clearly highlights the need for more benchmarking comparative studies to eventually contribute to a justified use of a specific WGS data analysis workflow within an international public health laboratory context.

## Introduction

Whole genome sequencing (WGS) is becoming increasingly recognized in a public health context as a single-shot method to determine species (Wood and Salzberg, 2014; Petersen et al., 2017), serotype (Joensen et al., 2015; Yoshida et al., 2016), antibiotic resistance (McDermott et al., 2016; Eyre et al., 2017) and virulence characteristics of pathogens (Schreiber et al., 2017; Mason et al., 2018). This technology also permits to most precisely determine the genetic differences between isolates, which are used for subtyping and to create phylogenies for surveillance and epidemiologic investigations of disease outbreaks (Qiu et al., 2015; Jackson et al., 2016; Durand et al., 2018). Because of the decrease in cost and turnaround time, WGS is becoming gradually more accessible for routine use in public health reference laboratories. In the last years, WGS has assisted the analysis of multiple bacterial outbreaks, some of which were international (Janmohamed et al., 2011; Launders et al., 2013; Inns et al., 2017; Schjørring et al., 2017; Genestet et al., 2019). Besides, several large national authorities are already applying WGS for routine surveillance and outbreak investigation of pathogens (ECDC, 2018; Painset et al., 2018; Rantsiou et al., 2018).

Despite the high potential to fulfill the needs of public health, there are hurdles that slow down the wide adoption of WGS for routine settings. One of such barriers is the complexity of WGS data analysis, including absence of a universal method suitable for all organisms and all applications, and difficulties to store and compare the obtained results. The most widely used approaches for extraction of high-resolution subtyping and relatedness information from WGS data can be grouped into methods based on core genome/whole genome multilocus sequence typing (cg/wgMLST), also termed gene-by-gene approaches, and methods based on single nucleotide polymorphism (SNP) detection (ECDC, 2016). The gene-by-gene approaches assess the diversity of isolates based on the alleles found for all (wgMLST) or core (cgMLST) genes of the species or genus of interest (Maiden et al., 2013). They rely on a database of all known allele variants for the selected genes, termed the subtyping scheme. Public cgMLST schemes have to be developed and curated for each organism separately, but a sustained and widely accepted scheme permits to store and exchange subtyping information between the laboratories worldwide (ECDC, 2016; Schürch et al., 2018). By contrast, SNP-based methods distinguish isolates based on SNPs present in the entire genome including the intergenic regions, potentially offering a higher resolution. SNP identification can be accomplished by the mapping of WGS reads to a reference genome, mutual alignment of *de novo* assembled sequences, or by counting short nucleotide sequences, kmers, in the raw or assembled data samples (Treangen et al., 2014). Establishment of a consistent nomenclature is inherently more complicated for all SNP-based subtyping approaches, and requires considerable standardization efforts to allow portability of the outcome. An example of such standardization effort is the system for defining and naming of clusters, named the ‘SNP address’ (Ashton et al., 2015). An important difference between the gene-by-gene and SNP-based approaches is the way they handle homologous gene transfer and other structural rearrangements that introduce polymorphism-rich stretches into the genome (Schürch et al., 2018). In SNP-based methods, regions containing high SNP densities as a result of recombination can provide misleading information about the genetic distances, interfere with reconstruction of ancestral sequences and potentially disturb the elucidation of clonal relationships between the isolates (Schierup and Hein, 2000a, b; Posada and Crandall, 2002; Croucher et al., 2011). Such regions are therefore often filtered out using specific recombination detection tools such as ClonalframeML (Didelot and Wilson, 2015) and Gubbins (Page et al., 2014). cgMLST methods on the contrary are relatively robust to such evolutionary events, collapsing regions with high SNP densities into a small number of allelic changes.

Despite the growing amount of comparative studies (de Been et al., 2015; Li et al., 2017; Ghanem and El-Gazzar, 2018; Halbedel et al., 2018; Janowicz et al., 2018; Meehan et al., 2018), it is currently often unknown which approach is the most suitable for a given organism and in a given epidemiological situation, and the dissimilarities in the output obtained using alternative approaches have been poorly characterized. This is a second problem that slows down the adoption of WGS in routine, i.e., the absence of an established methodology for the evaluation and comparison of WGS data analysis schemes. The different implementations of the methods described above can produce different outputs depending on the applied data processing steps and settings (Lüth et al., 2018; Saltykova et al., 2018). Studies which determine the performance characteristics such as reproducibility and discriminatory power of the WGS subtyping pipelines similarly to the classical methods, or that use various metrics to measure similarity between the SNP distance matrices and phylogenies generated by the different data analysis workflows have only recently started to emerge (David et al., 2016; Henri et al., 2017; Katz et al., 2017; Pearce et al., 2018; Saltykova et al., 2018). Other important features such as the stability of the data analysis workflows toward the characteristics of the input sequencing data, the effect of the reference genome used for subtyping on the output, or the suitability of a workflow for subtyping of isolates with a particular relatedness level, are currently rarely assessed.

**FIGURE 1:**
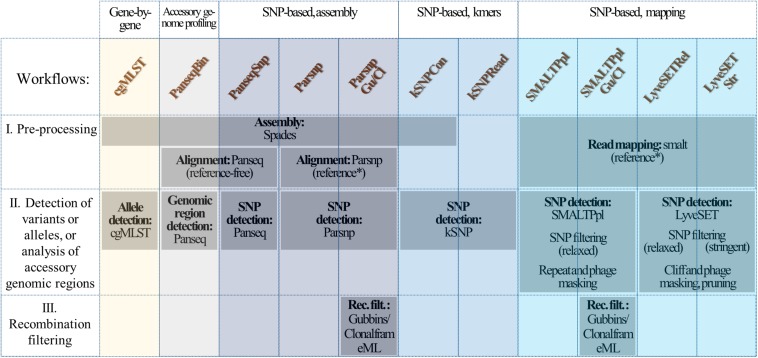
Subtyping workflows. Current study included the following subtyping workflows: a gene-by-gene workflow (cgMLST), a workflow based on accessory genome profiling (PanseqBin), assembly based workflows that used respectively Parsnp and Panseq for alignment of *de novo* assembled genomes and detection of polymorphisms (Parsnp and PanseqSNP), k-mer-based workflows using kSNP for SNP detection and either assembled contigs (kSNPCon) or reads (kSNPRead) as input, and mapping-based approaches, using either a combination of common tools for read mapping and SNP detection (SMALTPpl) or a high-quality SNP detection pipeline (LyveSETStr and LyveSETRel). PanseqBin, the approach based on accessory genome profiling, measures the differences between isolates based on presence or absence of accessory genomic regions. The two LyveSET-based workflows used different settings, namely in LyveSETStr, a relatively strict set of SNP filtering parameters is applied and in LyveSETRel, a relaxed SNP filtering is performed. Parsnp and SMALTPpl were also tested in combination with either ClonalframeML or Gubbins for filtering of recombinant regions (ParsnpCl/Gu and SMALTPplCl/Gu). Besides LyveSET and Panseq, all workflows have been previously used for subtyping of *N. meningitidis* (see Materials and Methods), in case of Parsnp and SMALTPpl in combination with either ClonalframeML or Gubbins. Panseq was included to represent an alternative assembly based approach and to evaluate accessory genome profiling for subtyping of *N. meningitidis*. It was used for characterization of other bacterial organisms marked by higher recombination or genomic rearrangement rates (Stewart et al., 2014; You et al., 2014; Delannoy et al., 2016; Kang et al., 2017). LyveSET was added to the comparison to represent a pipeline with extensive SNP filtering, which also includes the possibility for SNP pruning. ^∗^The reference-based pipelines were applied with a high-quality PacBio assembly of one of the isolates.

In this study we performed a comparison between the existing WGS workflows for subtyping of *Neisseria meningitidis*, the causative agent of meningococcal disease. This organism is marked by substantial homologous gene transfer, with the rates of nucleotide changes due to recombination exceeding those due to point mutation (Jolley et al., 2005). In the majority of the studies, subtyping of *N. meningitidis* is being carried out using a well-established cgMLST scheme, developed by Jolley et al. (2018). Recently, a number of works have also applied assembly-, kmer- and mapping-based SNP approaches in some cases combined with recombination detection tools, for characterization of *N. meningitidis* (Figure 1) (Lamelas et al., 2014, 2017; Mustapha et al., 2015; Sater et al., 2015; Stefanelli et al., 2016; Bårnes et al., 2017; Diallo et al., 2017; Tzeng et al., 2017; Hao et al., 2018; Whaley et al., 2018). However, it is not always that clear which method is the best to use for a given research question (Harrison et al., 2017; Whaley et al., 2018). Several studies demonstrated a high degree of agreement between the different SNP-based approaches and cgMLST on distinguishing epidemiologically linked isolates (Bårnes et al., 2017; Diallo et al., 2017; Whaley et al., 2018), but no detailed evaluation has been carried out describing the differences in performance and output of cgMLST and SNP-based workflows, and how they are affected by the use of tools that detect and mask recombinant regions. Given the exceptionally high rates of homologous gene transfer and structural variation, the variety of methods which have been used to subtype this organism, and the lack of a benchmarking analysis, *N. meningitidis* makes an interesting proof of concept to (a) evaluate the technical performance of the individual subtyping workflows, and recombination filtering tools and (b) to compare the output obtained with the different data analysis approaches.

We first compared the phylogenetic trees obtained with all tested workflows for the collection of isolates included in this study. Then, a more detailed evaluation of workflow performance was carried out using a selection of isolates belonging to clonal complex (cc) 269. Technical performance characteristics of the workflows were firstly assessed by calculating the performance metrics used for evaluation of classical subtyping methods, more specifically epidemiologic concordance, discriminatory power and reproducibility metrics (David et al., 2016). Secondly, robustness of the methods toward the variation of the input data was evaluated by assessing similarity of pipeline output obtained using replicate sequencing datasets and datasets with different coverage. For two of the more stably performing reference-based SNP-based workflows, the effect of the reference genome was evaluated. Upon evaluation of the technical performance of the workflows, the methods were compared between each other. Therefore, metrics described by Katz et al. (2017) were applied. Our results allowed to identify workflows with the most stable performance for *N. meningitidis* isolates of different levels of relatedness, and to point out which of the tested methodologies produced more similar genetic distances and phylogenies. While *N. meningitidis* was used for demonstration, we presented a strategy for a comparative benchmarking study which can be applied to the WGS data analysis tools used for other organisms.

## Materials and Methods

### Isolates, Genomic DNA Extraction and Sequencing Data Acquisition

For this study, we made use of a set of 69 isolates of *N. meningitidis*, chosen from the collection of 2002 to 2016 of the Belgian National Reference Centre (NRC) for *Neisseria* (Table 1). The selection contained a large portion of isolates from cc-269, which is the second most frequent cc for the B serogroup in Belgium, and is emerging in Europe during the last decade. Moreover, these isolates belong to the same cc and in some cases serosubtype, as B:NT:P1.14 isolates which were described in 2011 to form a clonal and endemic cluster in Belgium (Bertrand et al., 2011). Further, the selection included several common isolates of serogroups B, C, W, and Y belonging to different classical clonal complexes like to cc-41/44 and cc-11 in order to investigate their relatedness between each other and to the cc-269 isolates, and as background cases (Table 1). Classical subtyping data were provided by the NRC and determined as described by Bertrand et al. (2011). Designation of antigen gene alleles for *porA*, *porB*, and *fetA* genes were obtained with the *Neisseria* pipeline described by Bogaerts et al. (2019), by comparing assembled sequencing data to reference alleles downloaded from the *Neisseria* PubMLST^1^ database (Jolley et al., 2018).

**TABLE 1:** Isolates used in this study.

**Sample**	**LDs**	**SDs**	**B:NT cc-269**	**B:NT cc-269**	**Clonal**	**Sequence**	**Serogroup:serotype:**	**PorB**	**PorA**	**PorA**	**FetA**
**ID**			**LDs**	**SDs**	**complex (cc)**	**type (ST)**	**serosubtype**	**VR**	**VR1**	**VR2**	
2002-116	*				cc-213	213	B:NT:P1.14	ND	22	14	F5-5
2003-047	*				cc-162	162	B:NT:P1.14	149	22	14	F5-9
2004-065	*		*		cc-269	269	B:NT:P1.14	199	22	14	F5-1
2005-190	*				cc-18	2718	B:NT:P1.14	3	22	14	F5-2
2006-171	*		*		cc-269	2693	B:NT:P1.14	199	22	14	F5-1
2007-018	*		*		cc-269	2693	B:NT:P1.14	199	22	14	F5-1
2007-051	*		*		cc-269	269	B:NT:P1.14	199	22	14	F5-1
2007-080	*		*		cc-269	269	B:NT:P1.14	199	22	14	F5-1
2007-172	*				cc-18	858	B:NT:P1.14	ND	22	14	F3-1
2008-034	*		*		cc-269	269	B:NT:P1.14	199	22	14	F5-1
2008-060	*		*		cc-269	269	B:NT:P1.14	199	22	14	F5-1
2008-114	*		*		ND	ND	B:NT:P1.14	1462	22	14	F4-1
2008-120	*		*		cc-269	269	B:NT:P1.14	199	22	14	F5-1
2009-014	*		*		cc-269	269	B:NT:P1.14	199	22	14	F5-1
2009-020	*				cc-213	213	B:NT:P1.14	ND	22	14	F5-5
2009-098	*		*		cc-269	2693	B:NT:P1.14	199	22	14	F5-1
2009-105	*				cc-162	162	B:NT:P1.14	149	22	ND	F5-9
2010-129	*		*		cc-269	2693	B:NT:P1.14	199	22	14	F5-1
2011-004	*		*		cc-269	2693	B:NT:P1.14	199	22	14	F5-1
2011-005	*	*	*	*	cc-269	269	B:NT:P1.14	199	22	14	F5-1
2011-006	*	*	*	*	cc-269	2693	B:NT:P1.14	199	22	14	F5-1
2011-010	*		*		cc-269	2693	B:NT:P1.14	199	22	14	F5-1
2011-023	*	*	*	*	cc-269	ND	B:NT:P1.14	199	22	14	F5-1
2011-025	*	*	*	*	cc-269	269	B:NT:P1.14	199	22	14	F5-1
2011-027	*		*		cc-269	2693	B:NT:P1.14	199	22	14	F5-1
2011-042	*		*		cc-269	269	B:NT:P1.14	199	22	14	F5-1
2011-058	*	*	*	*	cc-269	269	B:NT:P1.14	199	22	14	F5-1
2011-086	*				cc-35	35	B:NT:P1.14	ND	22-1	14	ND
2012-079	*		*		cc-269	ND	B:NT:P1.14	199	22	14	F5-1
S13BD01093	*	*	*	*	cc-269	269	B:NT:P1.14	199	22	14	F5-1
S13BD02289	*		*		cc-269	269	B:NT:P1.14	199	22	14	F5-2
S15BD00757	*	*	*	*	cc-269	2693	B:NT:P1.14	199	22	14	F5-1
S15BD01319	*		*		cc-269	2693	B:NT:P1.14	199	22	14	F5-1
S15BD03615	*		*		cc-269	2693	B:NT:P1.14	199	22	14	F5-1
**S13BD00117**	*	*	*	*	**cc-269**	**269**	**B:NT:P1.5,2**	**199**	**5-1**	**2**	**F5-1**
**S13BD00431**	*	*	*	*	**cc-269**	**269**	**B:NT:P1.5,2**	**199**	**5-1**	**2**	**F5-1**
**S13BD00761**	*	*	*	*	**cc-269**	**269**	**B:NT:P1.5,2**	**199**	**5-1**	**2**	**F5-1**
**S13BD01533**	*	*	*	*	**cc-269**	**269**	**B:NT:P1.5,2**	**199**	**5-1**	**2**	**F5-1**
S13BD01748	*				cc-60	2209	B:NT:P1.5,2	389	5	2	F3-7
**S13BD02841**	*		*		**cc-269**	**269**	**B:NT:P1.5,2**	**199**	**5-1**	**2**	**F5-1**
**S13BD03579**	*		*		**cc-269**	**269**	**B:NT:P1.5,2**	**199**	**5-1**	**2**	**F5-1**
**S13BD03733**	*	*	*	*	**cc-269**	**269**	**B:NT:P1.5,2**	**199**	**5-1**	**2**	**F5-1**
S13BD03907	*		*		cc-269	269	B:NT:P1.5,2	199	5	2	F5-1
**S14BD01180**	*				**cc-269**	**269**	**B:NT:P1.5,2**	**199**	**5-1**	**2**	**F5-1**
**S14BD01880**	*		*		**cc-269**	**269**	**B:NT:P1.5,2**	**199**	**5-1**	**2**	**F5-1**
**S14BD04646**	*	*	*	*	**cc-269**	**269**	**B:NT:P1.5,2**	**199**	**5-1**	**2**	**F5-1**
S15BD00088	*	*	*	*	cc-269	269	B:NT:P1.5,2	199	5	2	F5-1
S15BD05018	*				NE	5063	B:NT:P1.5,2	473	5-1	2	F1-5
S15BD07026	*	*			NE	11959	B:NT:P1.5,2	2	5-1	9	F4-1
S15BD00217	*	*			cc-41/44	2925	B:4:P1.4	42	7-2	4	F1-5
S15BD02364	*				cc-41/44	41	B:4:P1.4	42	7-2	4	F1-5
S15BD06042	*	*			cc-41/44	41	B:4:P1.4	42	7-2	4	F1-5
2009-015	*				cc-11	247	W:2a:P1.5,2	1	5	2	F3-1
2012-152	*				cc-11	1025	W:2a:P1.5,2	244	5	2	F1-1
S13BD03226	*	*			cc-11	11	W:NT:P1.5,2	ND	5	2	F1-1
S14BD04865	*				cc-11	11	W:2a:P1.5,2	244	5	2	F1-1
S15BD01379	*	*			cc-11	11	W:2a:P1.5,2	244	5	2	F1-1
S15BD04089	*				cc-11	11	W:2a:P1.5,2	244	5	2	F1-1
S13BD03199	*				cc-11	11	C:2a:P1.5,2	1	5	2	F3-3
S13BD03739	*	*			cc-11	11	C:2a:P1.5,2	1	5	2	F3-3
S15BD02596	*	*			cc-11	11	C:2a:P1.5,2	1	5	2	F3-3
S15BD09234	*	*			cc-11	11	C:2a:P1.5,2	1	5	2	F3-3
S16BD01507	*				cc-11	11	C:NT:P1.5,2	1	5	2	F3-6
S16BD01540	*				cc-11	11	C:NT:P1.5,2	1	5	2	F3-6
2012-040	*				NE	5436	Y:NT:P1.3,6	257	18-1	3	F3-4
S13BD01417	*				NE	5436	Y:NT:P1.3,6	257	18-1	3	F3-4
S14BD01395	*				NE	5436	Y:NT:P1.3,6	257	18-1	3	F3-4
S14BD01857	*	*			NE	5436	Y:NT:NST	257	18-1	3	F1-2
S15BD05503	*	*			NE	5436	Y:NT:P1.3,6	257	18-1	3	F3-4

*Isolates belonging to the two large datasets (LDs) and the two small datasets (SDs), and to the B:NT cc-269 subsets of the LDs (B:NT cc-269 LDs) and SDs (B:NT cc-269 SDs) are indicated with asterisks. S14BD01180 was excluded from the LDs due to insufficient coverage in LD1. **Bold:** B:NT:P1.5,2 isolates with identical subtyping information, referred to as B:NT:P1.5,2^∗^. **Bold and underlined**: isolates with confirmed epidemiological link. ND, not determined; NT, non-serotypeable; NST, non-sero-subtypeable; NE, clonal complex does not exist. Clonal complex (cc), sequence type (ST), PorB VR, PorA VR1, PorA VR2, and FetA determined *in silico* as described in Bogaerts et al. (2019), Serogroup:serotype:serosubtype determined *in vitro* as described in Bertrand et al. (2011). In Bertrand et al. (2011), isolate 2008-114 was identified as ST2592.*

**FIGURE 2:**
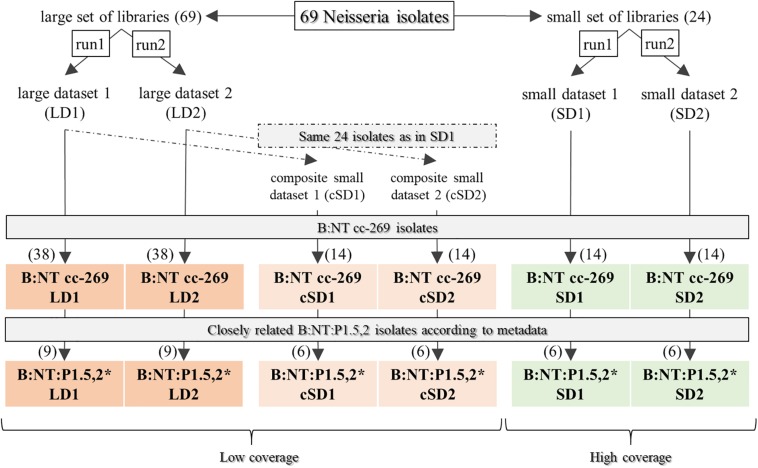
Datasets. From the original collection of 69 isolates (Table 1), two sets of Illumina libraries of 69 and 24 isolates respectively, were generated and sequenced yielding two pairs of replicate datasets with a different mean coverage: large dataset 1 (LD1) and large dataset 2 (LD2) originating from the 69-isolates library, and small dataset 1 (SD1) and small dataset 2 (SD2) originating from the 24-isolates library. Isolates that can be subtyped using a single reference genome were selected by KmerID, which attributed 38 isolates from the two LDs (referred to as B:NT cc-269 LD1 and B:NT cc-269 LD2), and 14 isolates from the SDs (B:NT cc-269 SD1 and B:NT cc-269 SD2) to the reference genome from clonal complex (cc) 269. The two cc-269 LDs and the two cc-269 SDs were used to evaluate pipeline stability toward inter-run variation. To evaluate the coverage sensitivity of the pipelines, composite small datasets (cSDs), were created, composed from data of the two LDs and containing the same 24 isolates as the SDs. Thereby, B:NT cc-269 cSDs were used as the low-coverage data, while the two B:NT cc-269 SDs represented the high-coverage data. The 38 B:NT cc-269 isolates (14 for SDs) selected for subtyping with the ST269 reference genome belonged to B:NT:P1.14 and B:NT:P1.5,2 subtypes (Table 1). Within the B:NT:P1.5,2 subtype, nine (six for SDs) isolates showed identical classical subtyping information, and three (two for SDs) of them were confirmed to be epidemiologically related by metadata. These nine (six for SD) B:NT:P1.5,2 isolates with identical classical subtyping information (referred to as B:NT:P1.5,2^∗^ in the main text), have been used as the group of related isolates during evaluation of epidemiologic concordance. Besides, as the B:NT:P1.5,2^∗^ isolates showed relatively small inter-isolate distances, they were used as the groups of closely related (outbreak) isolates during SNP matrix comparison tests.

From the collection of 69 isolates, two sets of Illumina libraries of 69 and 24 isolates were generated and sequenced yielding two pairs of replicate datasets with a different mean coverage: large dataset 1 (LD1) and large dataset 2 (LD2) originating from the pool of 69-isolates libraries, and small dataset 1 (SD1) and small dataset 2 (SD2) originating from the pool of 24-isolates libraries (Figure 2). Hereto, the isolates were grown on columbia-blood plates and genomic DNA was obtained from a single colony using Qiagen Genomic-tip 100/G kit (Qiagen) according to the manufacturer’s instructions. Two pools of Nextera sequencing libraries were prepared, with 69 and 24 isolates, respectively. Each library pool was sequenced twice on an Illumina MiSeq (Illumina Inc.) using the MiSeq Reagent Kit v3 (Illumina Inc.), obtaining 300 bp paired-end reads. Raw data was adapter-trimmed during demultiplexing with the Generate Fastq workflow (Illumina Inc.). The raw reads used in this study have been deposited in the NCBI Sequence Read Archive under accession number PRJNA549235.

Prior to running the workflows, the quality of the sequencing data was analyzed with FastQC 0.11.4 (Andrews, 2010), using MultiQC 1.6 (Ewels et al., 2016) to summarize the results. The number of reads per sample varied between 117111 and 671853 reads for LD1, 237217 and 1480998 reads for LD2, 450770 and 1735212 reads for SD1, and 255967 and 1086290 reads for SD2 (Supplementary Table S1). This corresponded to a mean coverage of 75X for LD1, 81X for LD2, 213X for SD1 and 146X for SD2 as determined using Qualimap 2.2.1 (Okonechnikov et al., 2016) based on reads mapped with SMALT 0.7.6 (Ponstingl and Ning, 2010) to a newly created PacBio reference genome (see below). One isolate, 14BD01180 could not be subtyped by cgMLST using data from LD1 because of a lower coverage (21X). This isolate was excluded from further analyses from both LDs, as it also caused a strong deterioration of the SNP matrix comparison results for some of the tested pipelines and was therefore considered to be non-typeable by these pipelines.

### Reference Genomes

Pacbio sequencing was carried out on a PacBio RS II instrument (Pacific Biosciences) at the Earlham Institute (Norwich, United Kingdom) using DNA of the isolate S13BD00117 obtained as described above. Raw sequencing data was pre-processed using SMRT analysis portal 2.3.0 (Pacific Biosciences), which yielded 47033 high-quality reads (320507566 bp) with a mean subread length of 6814 bp and N50 of 9216 bp. Processed reads were assembled with SMRT analysis 2.3.0, yielding two contigs, one of 2277000 bp, with a coverage of ∼125X and overlapping ends, and one of 14819 bp with a coverage of ∼5X. The small contig was discarded based on the coverage filter. Assembly was finished using minimus2 and fixstart functions from Circulator 1.4.0 (Hunt et al., 2015) for circularization and SMRT analysis 2.3.0 Assembly Polishing Module for final error correction.

Nanopore sequencing was carried out in-house on the same DNA extract as was used for PacBio sequencing, on a FLO-MIN106 R9.4 (FAF01498) flowcell (Oxford Nanopore Technologies) and using a LSK108 library prep kit (Oxford Nanopore Technologies) and a NBD103 barcode kit (Oxford Nanopore Technologies). Basecalling was performed with Albacore 2.0.1 (Oxford Nanopore Technologies) and demultiplexing and adapter trimming was carried out with Porechop^2^ 0.2.2, yielding 199278 high quality reads (1107136137 bp) with an average read length of 5555 bp and N50 of 7936 for the barcode of interest. A hybrid *de novo* assembly was carried out with SPAdes 3.11.1 (Bankevich et al., 2012) using Illumina sequencing data of the same isolate from SD1. Upon removal of contigs with a coverage < 5X, the assembly contained a single 2264862 bp contig with overlapping ends. The draft assembly was finished using minimus2 and fixstart functions from Circulator 1.4.0, and several rounds of Quiver (Chin et al., 2013) error correction with the available read data.

The two assemblies displayed 24 SNPs and 51 gaps with a maximal size of 52 bp (Supplementary Figure S1) between each other. Because of the high coverage, low error rate and long read length, the Pacbio assembly showed a very high quality, and the majority of the differences were likely due to errors of the Nanopore assembly.

### KmerID

Groups of closely related isolates that can be subtyped using the same reference genome were identified using KmerID^3^, a tool that determines similarity between sequencing samples and/or assemblies based on kmer content. The collection of reference genomes used in the analysis consisted of 14 reference genomes, and was created by selecting one reference per clonal complex from the 73 high-quality *N. meningitidis* assemblies available on NCBI. Isolates were attributed to the most closely related assembly if they demonstrated at least 85% similarity to it.

### Workflows

The workflows evaluated in this study, have been summarized in Figure 1. Prior to the analysis, a number of different parameter sets have been briefly evaluated for some workflows (e.g., the fragmentation size and the percentage identity cut-off for Panseq, and the minimal coverage for LyveSET) in order to choose the optimal configuration for the current study.

#### cgMLST

The tested implementation of the cgMLST approach is described in Bogaerts et al. (2019). It is based on the regularly updated cgMLST typing scheme maintained by the PubMLST platform (Jolley et al., 2018). Briefly, the tested pipeline consists of the following data analysis steps: raw reads are trimmed using Trimmomatic (Bolger et al., 2014) removing low-quality bases and clipping Illumina adapters, and *de novo* assembled using SPAdes. The contigs are aligned against the cgMLST database for sequence typing using blastn. The best hit for each cluster is determined by the method for allele scoring as described by Larsen et al. (2012).

In the current study, alleles identified by the pipeline for each of the 1605 tested genes and for each isolate were saved in an allele matrix. The matrix was filtered to remove genes for which no allele was called in more than 10% of the isolates and used for the downstream analyses.

#### Panseq: PanseqBin and PanseqSNP

The Panseq pipeline allows to identify the core and accessory genome among a collection of genomic sequences, find SNPs within the core genome and determine the presence/absence of genomic regions within the accessory genome (Laing et al., 2010). The raw sequencing data was assembled using SPAdes 3.11.1 at default settings. Panseq 3.2.1 was run at default parameters, including a minimum novel region size of 500, a fragmentation size 500, a percent identity cutoff of 85% and retaining positions present in 90% of the isolates. Additional fragmentation sizes of 100 and 300 bp, as well as percent identity cutoffs of 90 and 80% were tested to find an optimal workflow but none of the parameter sets showed an overall better performance than the default values (data not shown). The pipeline produced two multiple alignment matrices, one based on normal SNPs detected in the aligned regions (PanseqSNP), and one based on a binary matrix indicating the presence and absence of accessory genomic regions for each of the analyzed isolates (PanseqBin, from Panseq Binary). Both matrices were used in the downstream analyses.

#### Parsnp

The Parsnp pipeline is designed for microbial core genome alignment, SNP detection and filtering (Treangen et al., 2014). Unless specified otherwise, Parsnp 1.2 was run using the high-quality Pacbio reference genome to ensure optimal performance for the B:NT:P1.5,2 clade. It was applied at default parameters, which were previously used for subtyping of *N. meningitidis* (Bårnes et al., 2017; Tzeng et al., 2017), on sequencing data that was assembled using SPAdes 3.11.1 at default setting. Parsnp was tested with, and without the option enabling filtering of SNPs located in PhiPack (Bruen et al., 2006) identified regions of recombination, but the output appeared to be nearly identical (data not shown). Therefore, only the configuration without the recombination filtering option was included in the final comparison. A fasta matrix consisting of concatenated aligned regions was obtained with Harvesttools 1.2 (Treangen et al., 2014), and provided as input to ClonalframeML (ParsnpCl) or Gubbins (ParsnpGu), or filtered to retain positions that contain less than 10% of missing values and used in downstream analyses.

#### kSNP: kSNPCon and kSNPRead

The kSNP pipeline, allowing SNP discovery based on k-mer analysis (Gardner et al., 2015), has been applied for subtyping of *N. meningitidis* in various studies (Stefanelli et al., 2016; Diallo et al., 2017; Whaley et al., 2018). In this work, kSNP 3.0 was tested with assembled data (kSNPCon, from kSNP Contigs) and trimmed reads (kSNPRead, from kSNP Reads) as input. For kSNPCon, sequencing data was assembled using the same approach as for Panseq. For kSNPRead, input data was quality-trimmed using Trimomatic 0.3 using the following settings: ILLUMINACLIP:2:30, LEADING:30, TRAILING:30, SLIDINGWINDOW:4:20, MINLEN:23. Optimal kmer-size, 23 bp, was selected using Kchooser (Gardner et al., 2015). The pipeline was tested with the three available options for SNP matrix filtering (–core to retain positions that are present in all isolates, –min_frac 0.9 to keep positions that are present in more than 90% of the isolates and –all to retain all positions in the final SNP matrix). The –min_frac 0.9 parameter performed optimally for reproducibility, discriminatory power and distance matrix comparison (data not shown) and was retained for the final analysis.

#### SMALT-Based Pipeline: SMALTPpl

This workflow, named by us after the read mapping tool that is used in this workflow, has been applied previously in slightly different configurations for subtyping of various bacterial species including *N. meningitidis* (Croucher et al., 2011; Lamelas et al., 2014, 2017; Sater et al., 2015; Kwong et al., 2016; Mentasti et al., 2017; Hao et al., 2018). In the current version which is similar to the one used by Sater et al. (2015) and Lamelas et al. (2017), reads were mapped at default parameters using SMALT 0.7.6, SNP calling was performed using Samtools 1.8 (Li et al., 2009) and SNP filtering was carried out with Bcftools 1.8 (Li, 2011) retaining positions with a minimal quality of 30, a minimal allele frequency of 75% and a minimal depth of 5 reads. Unless otherwise specified, the Pacbio assembly was used as a reference genome. Two options of the pipeline were tested, one with and one without masking of phage regions detected using Phaster (Arndt et al., 2016), and repeats detected using repeat-match from MUMmer 3.23 (Kurtz et al., 2004) with a minimal repeat size of 50 bp. The version with masking performed slightly better for the SNP matrix comparison tests performed with replicate datasets (data not shown), and was retained for further analysis. The final SNP matrix was used as input for ClonalframeML (SMALTPplCl) or Gubbins (SMALTPplGu), or filtered to remove positions with more than 10% of missing values for downstream analyses.

#### LyveSET: LyveSETRel and LyveSETStr

LyveSET is a high-quality SNP pipeline which allows extensive quality filtering and pruning of SNPs (Katz et al., 2017). LyveSET 2.0.1 was run using the Pacbio reference genome, CPG read cleaner, an alternative allele frequency of 75%, and a minimal coverage of 10 reads as applied in the listeria_monocytogenes presets (LyveSETStr, from LyveSET Strict) or a minimal coverage of 5 reads as applied in SMALTPpl (LyveSETRel, from LyveSET Relaxed). Both pipeline variations were tested with and without masking of ‘cliff’ regions (Katz et al., 2017), pruning of SNPs that were located closer to each other than 100 bp, and filtering of phage regions. The pipelines that included all of these options produced more similar SNP distance matrices and phylogenetic trees with replicate datasets (data not shown) and were retained. The final SNP matrix was filtered to remove positions with more than 10% of missing values and used for downstream analyses.

#### ClonalframeML and Gubbins: SMALTPpl Gu/Cl and Parsnp Gu/Cl

To assess for recombination in the output of SMALTPpl and Parsnp, aligned genomic sequences and RAxML maximum-likelyhood phylogenetic trees were processed by ClonalframeML 1.11.3 (Didelot and Wilson, 2015). The tool was run under the standard model with 100 simulations (-emsim 100) and with the relative rate of transition versus transversion (kappa) determined by PhyML 3.1 (Guindon et al., 2010) at default parameters. Additional assessment of recombination in the aligned genome sequences was performed with Gubbins 2.2.1 (Page et al., 2014), which was run at default parameters with maximum 15 iterations. The output of ClonalframeML and Gubbins was processed using maskrc-svg^4^, to obtain the aligned genome sequences with masked recombinant regions. Generated fasta matrices were filtered, retaining sites with less than 10% of missing values, and used for downstream analyses.

#### Phylogenetic Trees and Distance Matrices

Distance matrices were created by comparing the allele/SNP/binary matrix positions for each isolate pair ignoring positions with missing values and counting positions with unequal values. Prior to construction of phylogenetic trees all allele/SNP/binary matrices were filtered to retain only informative positions with snp-sites 2.4.1 (Page et al., 2016). The phylogenies were inferred with RAxML 8.2.11 (Stamatakis, 2014), using GTRGAMMA model for the fasta matrices and BINGAMMA model for the binary matrices generated by PanseqBin, with 100 bootstrap replicates and performing a rapid bootstrap analysis and search for best-scoring ML tree in one program run. For the cgMLST phylogenetic tree, as well as the UPGMA trees generated for the SNP-based pipelines, the relative distances between the isolates were calculated as the number of genetic distances that are different between the isolates divided by the total number of genes or genomic positions shared by the two isolates, upon which phylogenetic tree construction was carried out with DistanceTreeConstructor UPGMA method of the Biopython-1.72 Phylo.TreeConstruction package (Talevich et al., 2012).

### Evaluation of Pipeline Performance

#### Epidemiologic Concordance, Discriminatory Power and Reproducibility

Epidemiologic concordance, defined as the fraction of pairs of epidemiologically related isolates that were assigned to the same type (David et al., 2016), was calculated as the Wallace’s coefficient with the only subtyping category being the outbreak isolates (cfr. Table 1) (Carrico et al., 2006). The discriminatory power of a typing scheme is its ability to discriminate between unrelated strains (Struelens, 1998). The index of discriminatory power (D) was calculated as Simpson’s index of diversity (Hunter and Gaston, 1988; Grundmann et al., 2001). NGS-based subtyping techniques will often detect some small differences between replicate sequencing samples of the same isolate. In case isolates are assigned to different subtypes as soon as they show a non-zero genetic distance, this can lead to an over-estimation of the discriminatory power (David et al., 2016; Saltykova et al., 2018). To avoid that, we have chosen to assign isolates to different subtypes as soon as they show higher distances between each-other than the maximal genetic distances observed between replicate sequencing samples of the same isolate.

Another important metrics to evaluate the accuracy of a subtyping scheme is reproducibility, which denotes the ability of a subtyping method to assign isolates to the same subtype during repetitive testing (Struelens, 1998). However, if the threshold for discrimination between pipelines is set based on genetic distances observed between replicate isolates, reproducibility will always equal 1. Therefore, we evaluate the reproducibility of a pipeline based on the maximal genetic distances observed between replicate isolates (which is the same as the threshold for discrimination between subtypes). The genetic distances between replicate isolates were determined as follows: five cc-269 isolates, S15BD00757, S13BD03733, S13BD00117, S13BD00761 and 2011-025, were chosen randomly. The sequencing data of the five isolates was exchanged between the replicate datasets of the same coverage, i.e., between LD1 and LD2; and SD1 and SD2, the analysis was rerun. The genetic distance threshold for each dataset was set to the maximal allele/SNP/binary distance observed between any of the five replicate pairs.

#### Comparison of Distance Matrices and Phylogenetic Trees

Similarity between distance matrices was assessed according to the compareSnps.sh^5^ script from Katz et al. (2017), allowing any distances between closely related isolates. Firstly, SNP distance matrices were compared by linear regression analysis, carried out with lm function from R 3.4.2 at default parameters. Output of the linear regression test includes slope and *r*^2^ values. Slope is the number of genetic differences in the query matrix that correspond to one genetic change in the reference matrix and *r*^2^ reflects the percentage of variation that is explained by the fitted model. Secondly, a Mantel test from R package Vegan (Dixon, 2003) was carried out, measuring the Spearman correlation between two distance matrices (Smouse et al., 1986).

Comparison of the phylogenetic trees was carried out according to compareTrees.sh^6^ script from Katz et al. (2017), allowing nodes with any bootstrapping values. Phylogenetic trees were rooted using S15BD00088 as an outgroup isolate, and compared with Kendall-Colijn test with λ = 0 (Kendall and Colijn, 2015) implemented in R package Treespace^7^. The test compares two trees using Euclidean distances from tip to root, with the coefficient λ allowing to give more weight to topology (λ = 0) or branch length (λ = 1). To estimate significance values, background distribution of 10^5^ random trees was created using the tree function from APE package in R 3.4.2 (Paradis et al., 2004). Thereby, the tree that is used as a query tree is compared to the tree that represents the reference tree and to the background distribution. A *z*-test is performed comparing the distance observed between the query and the reference to the distances observed between the query and the trees from the random distribution. A *p*-value lower than 0.05 indicates that the query tree is more closely related to the reference tree topology than would be expected by chance.

## Results

### WGS-Based Investigation of Relatedness Between *N. meningitidis* Isolates

**FIGURE 3:**
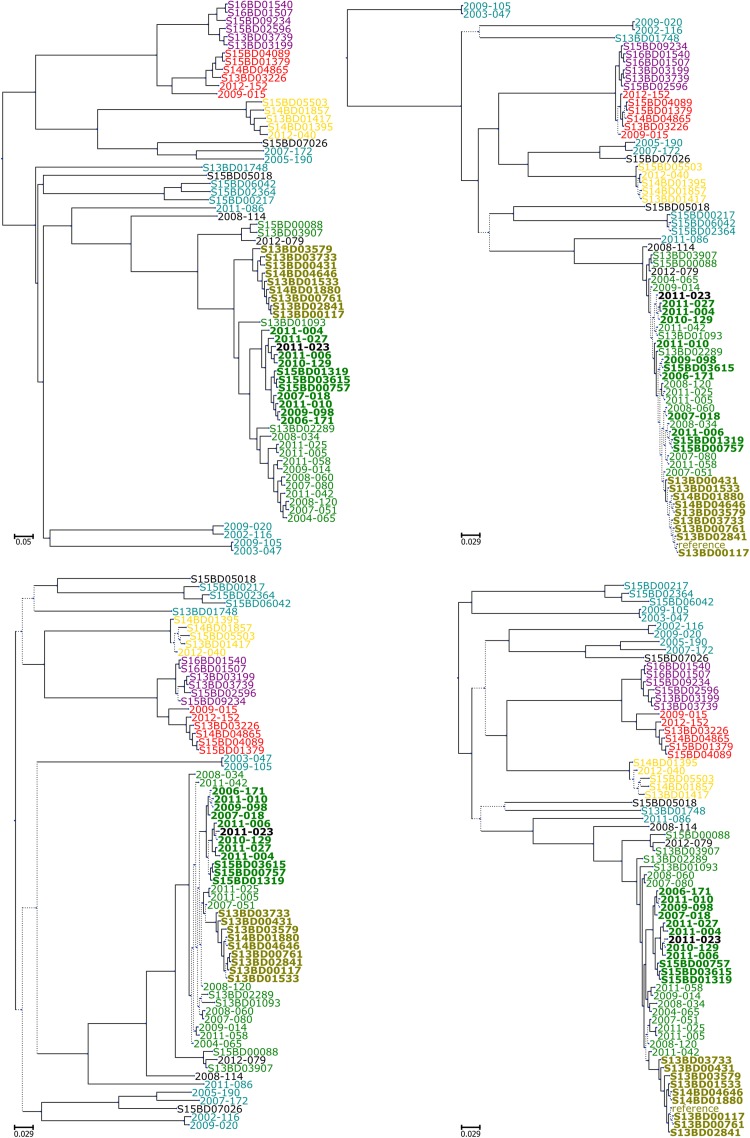
Initial comparison of the subtyping workflows. **Upper left panel:** phylogenetic tree generated with cgMLST. **Upper right panel:** phylogenetic tree generated with PanseqGu. **Lower left panel:** phylogenetic tree generated with kSNPRead. **Lower right panel:** phylogenetic tree generated with SMALTPpl. All phylogenetic trees were obtained using the large dataset 1 (LD1). For the SMALTPpl **(lower right panel)** and ParsnpGu **(upper right panel)** workflows, PacBio was used as reference genome. Branches with support values lower than 70% are displayed as dotted lines. Color codes: **red:** serogroup W, **yellow:** serogroup Y, **purple:** serogroup C, **green:** serogroup B isolates belonging to cc-269 clonal complex except one sub-branch, B:NT:P1.5,2^∗^, which is colored **olive**, **cyan:** serogroup B isolates belonging to other clonal complexes than cc-269, **black:** serogroup B isolates with undetermined clonal complex. Two clades of cc-269 isolates discussed in more detail in the main text, B:NT:P1.14 ST2693 and B:NT:P1.5,2^∗^ are indicated in bold.

In the phylogenetic trees generated by all tested workflows (Figure 1) using the original sequencing datasets (LD1, LD2, SD1, and SD2, Figure 2), all cc-269 isolates and three isolates with non-determined cc formed a monophyletic group (Figure 3 and Supplementary Data Sheet S1). Within the group, two additional clades of more closely related isolates were present in most phylogenies. The first consisted of 11 B:NT:P1.14 ST2693 isolates and one B:NT:P1.14 isolate with an undetermined ST obtained between 2006 and 2015. The second contained 9 B:NT:P1.5,2 ST269 isolates harboring for PorA the allele 5-1 for VR1 that emerged in 2013 and 2014, including three isolates confirmed to be epidemiologically related by metadata. Further, cc-11 isolates were placed to a separate clade by all workflows, with two sub-branches corresponding to serogroups W and C. Additional stably recurring isolate associations identified by all workflows and with all datasets included the serogroup Y isolates, and the non-cc-269 serogroup B isolates belonging to a common clonal complex, i.e., cc-18 (isolate ID: 2005-190 and 2007-172), cc-213 (isolate ID: 2002-116 and 2009-020) and cc-162 (isolate ID: 2003-047 and 2009-105). The relative positions of these commonly retrieved groups within the phylogenetic trees, however, differed considerably between the workflows and datasets, therefore not allowing to make a conclusion on their relation with cc-269. Notably, according to most pipelines, the B:NT:P1.14 isolates belonging to the different clonal complexes were often not more closely related to each other than to the B:4:P1.4 isolates included as background cases (isolate ID: S15BD00217, S15BD02364 and S15BD06042). The only exception to that was the B:NT:P1.14 isolate belonging to cc-35 (isolate ID: 2011-086), that was consistently placed at the root of the cc-269 branch by all workflows and with all datasets.

The obtained results showed that while all of the workflows retrieved the same groups of genetically similar isolates, the relations between these groups, and the corresponding genetic distances differed between the workflows. In the subsequent analyses, the sequencing data from the two LDs and the two SDs were used to assess the performance of the pipelines and their sensitivity toward variation of input sequencing data and to compare the output between the pipelines (Figure 2 and Table 1). In addition, a pair of low-coverage cSDs datasets containing the same isolates as SDs were composed using sequencing data from LDs to evaluate pipeline sensitivity to coverage changes. Because high-resolution subtyping is preferably carried out on a set of more closely related isolates (David et al., 2016), e.g., isolates belonging to the same cc, all tests were performed using subsets of the current isolate collection containing isolates that show more than 85% similarity to each other and to the ST269 reference genome as determined by KmerID (Figure 2 and Table 1). The created subsets included all the cc-269 isolates and the three isolates with undetermined cc that were attributed to the same cluster on the phylogenetic trees (further called B:NT cc-269). Thereby, a subgroup of the cc-269 isolates, namely the 9 B:NT:P1.5,2 isolates harboring the VR1 5-1 allele for PorA (further called B:NT:P1.5,2^∗^), was used as the cluster of epidemiologically related isolates during evaluation of epidemiologic concordance, and as the group of closely related isolates with small inter-isolate distances during SNP matrix comparison tests.

### Technical Performance Characteristics of Subtyping Workflows

#### Concordance, Discriminatory Power, Reproducibility and SNP Matrix Size

**FIGURE 4:**
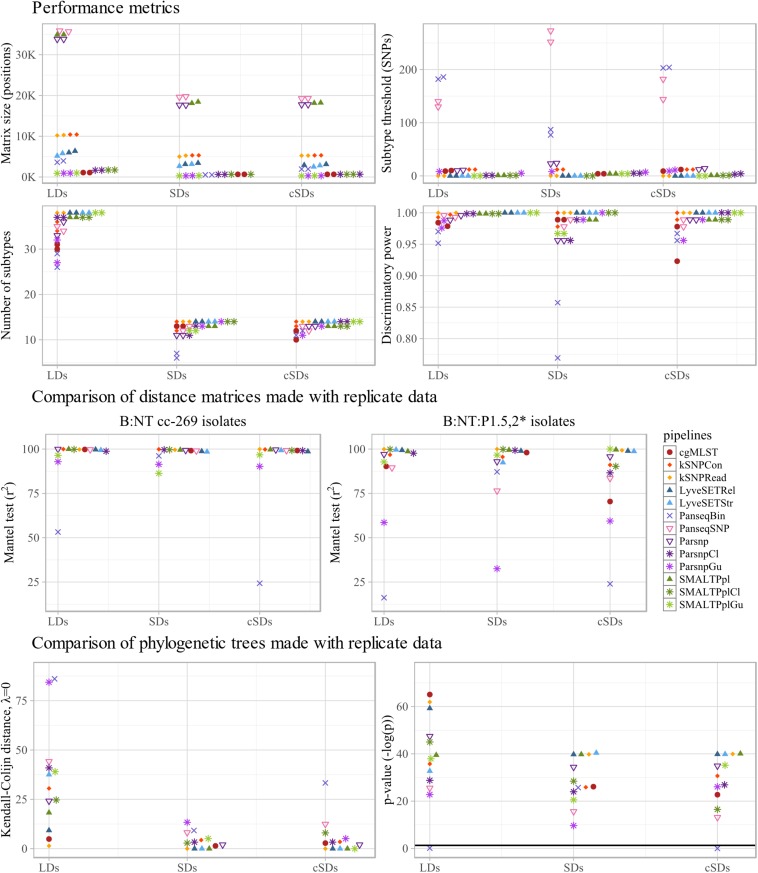
Technical performance characteristics of subtyping workflows. **Upper four panels:** performance metrics of the tested pipelines, more specifically SNP/allele matrix size, threshold for differentiation between subtypes, the number of subtypes discriminated by the pipelines and the corresponding discriminative power (D) are shown. For each metric, the values calculated with the two large datasets (LDs), two small datasets (SDs) and two composite small datasets (cSDs) are displayed separately along the x-axis. **Middle two panels:** to assess stability of each pipeline to inter-run variability of sequencing data, distance matrices produced with replicate sequencing datasets were compared using Mantel test. The comparison was carried out between the two large datasets (LDs), the two small datasets (SDs), and the two composite small datasets (cSDs) (indicated on the *x*-axis), using all B:NT cc-269 isolates **(left)**, as well as a subset of more closely related isolates, B:NT:P1.5,2^∗^ **(right)**, and the resulting Spearman correlation coefficients (*r*^2^) were reported. **Lower two panels:** to further assess stability of each pipeline to inter-run variability of sequencing data, phylogenetic trees produced with replicate sequencing datasets were compared using the Kendall–Colijn (KC) test for topology (λ = 0). The comparison was carried out between the two large datasets (LDs), the two small datasets (SDs), and the two composite small datasets (cSDs) (indicated on the *x*-axis), and the resulting KC values **(right)** and the corresponding *p*-values, expressed as -log(p) **(left)**, were shown. Thick horizontal line corresponds to *p* = 0.05 threshold.

The different performance metrics of the workflows were determined based on B:NT cc-269 LD1, B:NT cc-269 LD2, B:NT cc-269 SD1 and B:NT cc-269 SD2 (Figure 4 upper panel and Supplementary Table S2). All workflows ascribed all of the B:NT:P1.5,2^∗^ isolates to a separate clade with all datasets, yielding a concordance of 100%. As expected, allele/SNP matrix sizes differed considerably between the pipelines, with the SMALTPpl, Parsnp and PanseqSNP detecting the largest number of polymorphisms, followed by kSNP- and LyveSET-based workflows, PanseqBin and cgMLST (Figure 4 upper panel, Matrix size). For the calculation of discriminatory power, the threshold for discrimination between subtypes was set equal to the genetic distances observed between replicate sequencing samples. The same value also reflects the reproducibility of the pipelines. kSNPRead and the three mapping-based subtyping workflows, LyveSETRel, LyveSETStr and SMALTPpl, showed relatively low SNP distances between replicate isolates and a high discriminatory power (D) (Figure 4 upper panel, Subtype threshold, Number of subtypes and Discriminatory power). Unlike kSNPRead, kSNPCon returned non-zero SNP distances between replicates, and an intermediate D, as did the two assembly based workflows, Parsnp and PanseqSNP. Concordantly with the smallest matrix size, the cgMLST pipeline showed a lower D, at least with LDs. Panseq-based pipelines generally reported the worst reproducibility, and PanseqBin also displayed the lowest D of all tested workflows.

Parsnp and SMALTPpl were tested with recombination filtering tools ClonalframeML and Gubbins (Figure 4 upper panel). Application of these tools drastically decreased the number of polymorphic sites, retaining less than 5% of SNPs with any of the datasets, with Gubbins performing a more stringent filtering than ClonalframeML (Figure 4 upper panel, Matrix size). Decrease of the SNP distances between isolates also decreased the number of SNPs observed between replicate isolates. For ClonalframeML, it resulted in some cases in an improvement of the D, while for Gubbins both improvement and decrease of D was observed depending on the pipelines and dataset (Figure 4 upper panel, Subtype threshold, Number of subtypes and Discriminatory power).

#### Stability Toward Inter-Run Variation of Input Sequencing Data

Further, the robustness of the workflows toward variation of the input data from replicate sequencing datasets was evaluated. Therefore, the pairwise distance matrices and phylogenetic trees produced with data from the two B:NT cc-269 LDs or the two B:NT cc-269 SDs, were compared (Figure 4 middle and lower panels and Supplementary Tables S3–S5).

The distance matrix comparison was performed using the Mantel test assessing Spearman’s rank-order association (Figure 4 middle panel and Supplementary Table S3) and linear regression analysis (Supplementary Table S4), and was carried out firstly with all the B:NT cc-269 isolates and secondly with the subset of B:NT:P1.5,2^∗^ isolates from the LDs and SDs, the latter allowing to consider the performance of the workflows with more closely related isolates. The Mantel test showed that all of the SNP-based workflows that do not use recombination filtering (i.e., PanseqSNP, Parsnp, kSNP-based pipelines, SMALTPpl, and LyveSET-based pipelines) and cgMLST produced highly correlated distance matrices with B:NT cc-269 isolate subsets from the replicate datasets (*r*^2^ > 98.6, Figure 4 middle panel, B:NT cc-269). PanseqBin returned unsatisfactory output, but only for the low-coverage datasets (*r*^2^ = 53.2 for LDs). These observations were largely confirmed by the output of the linear regression analysis (Supplementary Table S4). An additional difference detected with the linear regression was that LyveSET-based pipelines, especially LyveSETStr, consistently demonstrated lower slope values from what was observed for other pipelines and from what is expected with replicate data (slope < 0.930 for LyveSETRel and slope < 0.876 for LyveSETStr compared to e.g., slope = 1.002 for cgMLST SDs). Also kSNPRead produced slightly lower slope values, but only with the SDs (slope = 0.948).

With the more closely related B:NT:P1.5,2^∗^ isolates, the most correlated pairwise distances according to the Mantel test were produced by the pipelines that use non-assembled reads as input, namely kSNPRead, SMALTPpl and LyvesetRel (*r*^2^ > 98.7 for LDs and SDs, Figure 4 middle panel, B:NT:P1.5,2^∗^), while cgMLST, PanseqSNP, Parsnp, kSNPCon and LyveSETStr demonstrated less correlated output (*r*^2^ < 95.6 for SDs or LDs). Linear regression analysis showed similar results, although the decline of Parsnp and kSNPCon performance with the closely related isolates was much less pronounced (Supplementary Table S4). The LyveSET-based workflows again showed lower than expected slope values.

The Mantel test results indicated that the use of Gubbins resulted in noticeably less correlated pairwise SNP distances between replicate datasets for SMALTPplGu and ParsnpGu pipelines both with B:NT cc-269 isolates and with B:NT:P1.5,2^∗^ isolates alone (96.6 ≥ r^2^ ≥ 32.5, Figure 4 middle panel, B:NT cc-269 and B:NT:P1.5,2^∗^). According to the same test, ClonalframeML had no large effect on the stability of the workflows for B:NT cc-269 isolates, and even improved the output slightly for B:NT:P1.5,2^∗^ isolates (Figure 4 middle panel). Linear regression analysis, however, showed that the output of ClonalframeML-based pipelines was more variable than that of the original pipelines with the B:NT:P1.5,2^∗^ isolates, with more deviating r^2^ and slope values with some of the datasets (Supplementary Table S4).

Similarity of the phylogenetic trees was measured using the Kendall-Colijn (KC) test for phylogenetic tree topology (λ = 0) (Figure 4 lower panel and Supplementary Table S5). All pipelines except PanseqBin produced phylogenetic trees that were more similar to each other than what would be expected by chance (*p* < 0.05) with both B:NT cc-269 LDs and B:NT cc-269 SDs (Figure 4 lower panel, *p*-value). The pipelines that do not use recombination filtering showed a variable performance depending on the datasets, but the KC distances between phylogenetic trees returned by PanseqBin and PanseqSNP were among the largest, while those returned by cgMLST, kSNPRead, LyveSETRel, and SMALTPpl – among the smallest with both LDs and SDs (Figure 4 lower panel, Kendall-Colijn distance). Pipelines utilizing Gubbins and ClonalframeML often produced topologically less similar trees with the replicate datasets compared to the original pipelines (Figure 4 lower panel, Kendall–Colijn distance and *p*-value).

Similarly to the phylogenetic trees obtained with all isolates, visual examination of the phylogenetic trees produced with the B:NT cc-269 LDs (Supplementary Data Sheet S1) showed that all pipelines except ParsnpGu stably retrieved two recurrent clades consisting of the B:NT:P1.5,2^∗^ and B:NT:P1.14 ST2693 isolates, respectively. The differences between the trees produced with replicate datasets were mainly in the location of the remaining B:NT:P1.14 ST269 isolates, as well as the positions of the more closely related isolates within the two stably recurring clades. Noticeably, SMALTPpl and kSNPRead produced identical arrangement of the B:NT:P1.5,2^∗^ isolates between the datasets, and placed the three confirmed epidemiologically related isolates together with all datasets, which was not the case for the other workflows. This observation is concordant with the combined results of Mantel test and linear regression analysis, according to which the two pipelines returned both highly correlated, and linear pairwise distances between the B:NT:P1.5,2^∗^ subsets of the two SDs and the two LDs (Figure 4 middle panel and Supplementary Tables S3, S4).

#### Sensitivity of the Workflows Toward Reference Genome

**FIGURE 5:**
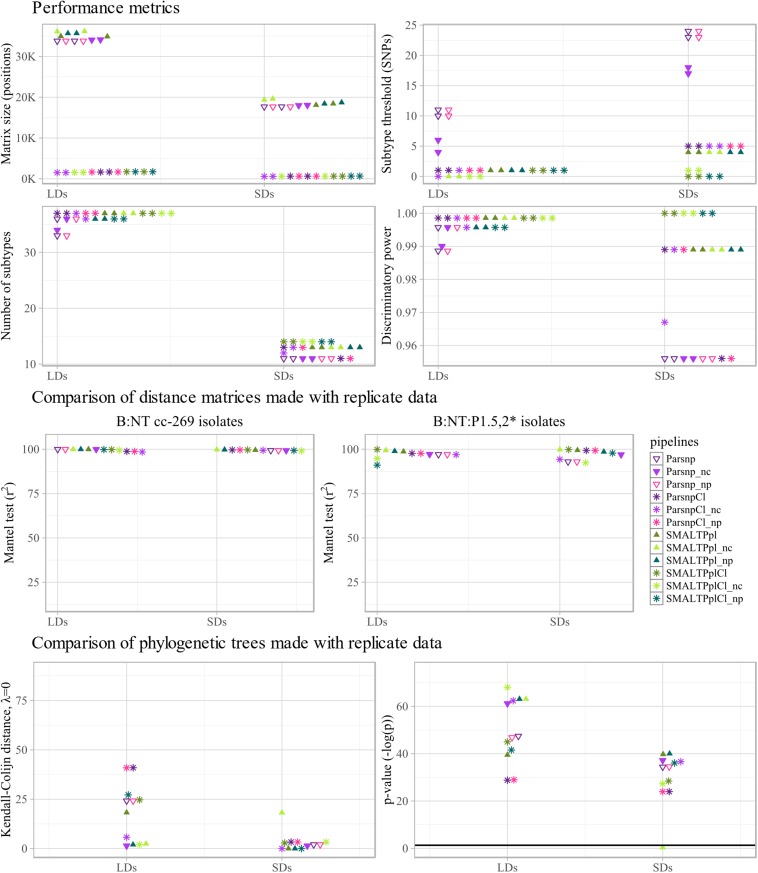
Technical performance characteristics of subtyping workflows: reference genomes. Performance of selected pipelines, i.e., Parsnp, SMALTPpl, ParsnpCl and SMALTPplCl, with three different reference genomes, more specifically Pacbio (/), hybrid Nanopore (np) and NC017515.1 (nc) was evaluated. **Upper four panels:** performance metrics of the tested pipelines, more specifically SNP/allele matrix size, threshold for differentiation between subtypes, the number of subtypes discriminated by the pipelines and the corresponding discriminative power (D) observed with the different reference genomes are shown. For each metric, the values calculated with the two large datasets (LDs), two small datasets (SDs) are displayed separately along the *x*-axis. **Middle two panels:** to assess how stability of each pipeline toward inter-run variability of sequencing data is altered by the reference genomes used, distance matrices produced with replicate sequencing datasets were compared using Mantel test. For each combination of pipeline and reference genome, the comparison was carried out between the two large datasets (LDs), and the two small datasets (SDs) (indicated on the *x*-axis), using all B:NT cc-269 isolates **(left)**, as well as a subset of more closely related isolates, B:NT:P1.5,2^∗^ **(right)**. The resulting Spearman correlation coefficients (*r*^2^) were reported. **Lower two panels:** to further assess how stability of each pipeline toward inter-run variability of sequencing data is altered by the reference genomes used, phylogenetic trees produced with replicate sequencing data are compared using the Kendall–Colijn (KC) test for topology (λ = 0). For each combination of pipeline and reference genome, the comparison was carried out between the two large datasets (LDs), and the two small datasets (SDs) (indicated on the *x*-axis), and the resulting KC values **(left)** and the corresponding *p*-values, expressed as –log(p) **(right)**, were shown. Thick horizontal line corresponds to *p* = 0.05 threshold.

To ensure optimal performance, the two mapping-based pipelines, SMALTPpl and LyveSET, as well as the assembly based Parsnp were applied with a high-quality circular reference genome assembled using PacBio sequencing data of one of the epidemiologically related B:NT:P1.5,2 isolates (S13BD00117, present in both LDs and SDs). In practice, rapid generation of a high-quality closely related PacBio assembly might not be possible and/or financially justified, and the analysis might have to be performed using an often more distinct publicly available reference. Alternatively, a closely related reference genome of a potentially lower quality could be generated using Nanopore sequencing technology, which is more accessible for smaller laboratories. In the current section, we have tested whether the source of the reference genome, and its relatedness to the isolates affected the performance of one mapping-based (SMALTPpl) and one assembly based (Parsnp) pipeline that showed a good performance in the previous tests. Also the combination of the two workflows with the more stable recombination-filtering tool, ClonalframeML, was analyzed (ParsnpCl and SMALTPplCl). Therefore, a second reference genome was generated for the S13BD00117 isolate using MinION and Illumina sequencing data, and a third high-quality reference genome of a rare isolate, B:NT:P1.19,15 also belonging to ST269 (NC017515.1, Budroni et al., 2011), was retrieved from NCBI. As in the previous section, performance was assessed based on performance metrics and the similarity of the SNP distance matrices and phylogenetic trees generated with the replicate sequencing datasets (Figure 5 and Supplementary Tables S6–S9).

For all of the tested pipelines, Pacbio and hybrid Nanopore references returned highly similar SNP matrix sizes, reproducibility and D, while the results obtained with the NC017515.1 reference genome were slightly more distinct, but lay in the same range for all three references (Figure 5 upper panel and Supplementary Table S6).

According to the Mantel test, SMALTPpl showed an equally stable performance with all three reference genomes and all dataset and data subset combinations (*r*^2^ > 98.6, Figure 5 middle panel, B:NT cc-269 and B:NT:P1.5,2^∗^, Supplementary Table S7). Parsnp, ParsnpCl and SMALTPplCl also demonstrated similar performance with the three different references with B:NT cc-269 isolates (*r*^2^ > 98.5). These results were confirmed by the linear regression analysis, with the only exception being slightly more deviating slope values observed with the NC017515.1 genome for ClonalframeML-based pipelines (Supplementary Table S8). For the more closely related B:NT:P1.5,2^∗^ isolates, however, Parsnp, ParsnpCl and SMALTPplCl demonstrated highly variable output with the different references according to the Mantel test (99.9 ≤ *r*^2^ ≤ 91.1 Figure 5 middle panel, B:NT:P1.5,2^∗^) and linear regression analysis (Supplementary Table S8). Because none of the references resulted in a clearly better output for any of the pipelines, this effect was likely due to the generally less stable performance of the workflows with closely related isolates which was demonstrated in the previous section.

The tested pipelines tended to produce more similar phylogenetic trees with replicate data if NC017515.1 was used as a reference genome, except for SMALTPpl which generated highly distinct phylogenetic trees with the two SDs (*p* < 0.05, Figure 5 lower panel, Kendall–Colijn distance and *p*-value, Supplementary Table S9). Visual examination of the two trees indicated that both contained identical clusters, and that the differences arose because of the arrangement of the clusters relative to each-other (Supplementary Data Sheet S1). Notably, for SMALTPpl (but not SMALTPplCl), the arrangement of the closely related B:NT:P1.5,2^∗^ isolates was the same for both LDs with all three reference genomes (Supplementary Data Sheet S1).

#### Sensitivity of the Workflows Toward Sequencing Coverage

Further, the sensitivity of the pipelines to sequencing coverage was evaluated using B:NT cc-269 SDs as high-coverage data and B:NT cc-269 cSDs as low-coverage data (Figure 2). Direct comparison between the SNP distance matrices and phylogenetic trees produced with B:NT cc-269 SDs and B:NT cc-269 cSDs was not performed, as thereby the effect of the coverage differences would be confounded with the effect of the inter-run variability between the high- and the low-coverage data. Instead, we have examined whether the pipeline performance, described using performance metrics and SNP distance matrix and phylogenetic tree comparison tests with replicate sequencing data, differed between high-coverage input (B:NT cc-269 SDs) and low-coverage input (B:NT cc-269 cSDs) (Figure 4, SDs vs. cSDs, Supplementary Tables S2–S5).

The strongest coverage sensitivity was demonstrated by PanseqBin, which showed less similar genetic distances and phylogenetic trees with the low-coverage data compared to high-coverage data (Figure 4 middle and lower panels, SDs vs. cSDs, Supplementary Table S4). Besides, PanseqBin detected larger distances between replicate isolates, and showed a large D with the low-coverage data (Figure 4 upper panel, Subtype threshold and Discriminatory power, SDs vs. cSDs). Also cgMLST appeared to be affected by the coverage changes, showing a lower reproducibility and D and less correlated and less linear allelic distances between closely related isolates with low-coverage input (Figure 4 upper and middle panels, SDs vs. cSDs, Supplementary Table S4). Other pipelines were less sensitive to the coverage of the input data, with no large differences observed between cSDs and SDs (Figure 4, SDs vs. cSDs, Supplementary Table S4).

Noticeably, ClonalframeML-based workflows showed worse Mantel test and linear regression results for the B:NT:P1.5,2^∗^ isolates with low-coverage data compared to high-coverage data (Figure 4 middle panel, B:NT:P1.5,2^∗^, SDs vs. cSDs, Supplementary Table S4). Also pipelines with Gubbins demonstrated different results with the low- and the high-coverage data for SNP matrix and phylogenetic trees comparison tests (Figure 4 middle and lower panels and Supplementary Table S4). However, the observed variation presented no clear trend, e.g., with low-coverage data Gubbins-based pipelines generated similar or slightly better Mantel test results with B:NT cc-269 isolate subsets, worse Mantel test results with the more closely related isolates, and similar or worse KC test results. Regarding the more variable results demonstrated by Gubbins pipelines with replicate sequencing datasets in the previous section, the observed variation could be a result of the generally unstable performance of the tool.

### Comparison of the Pipeline Output Using Statistical Tests

The second part of the analysis consisted of the mutual comparison of the subtyping workflows, evaluating whether they produce correlated and linear pairwise genetic distances between isolates, and topologically similar phylogenetic trees.

#### Genetic Distance Matrix Comparison

The Mantel test performed using B:NT cc-269 subsets of LDs and SDs illustrated that between each-other, the tested pipelines produced more correlated genetic distances according to the used recombination-filtering strategy, i.e., pipelines in which recombination filtering was omitted (including cgMLST), pipelines containing ClonalframeML, and pipelines containing Gubbins tended to form three separate clusters (respectively *r*^2^ > 94.6, *r*^2^ > 98.6 and *r*^2^ > 81.1 for the three clusters with LDs and SDs, Supplementary Figures S2A, S3A). A partial exception to that was LyveSETStr, which clustered slightly closer to ClonalframeML with one of the two SDs (Supplementary Figure S3A). Among the pipelines that used no recombination filtering, SMALTPpl, kSNP-based pipelines and Parsnip stably produced mutually highly correlated genetic distance matrices, as did LyveSET-based workflows, with *r*^2^ values in the same range as observed between replicate datasets (*r*^2^ > 98.6 for LDs and SDs). cgMLST demonstrated the highest correlation with SMALTPpl (*r*^2^ > 97.3 for LDs and SDs), kSNP-based pipelines (*r*^2^ > 96.8 for LDs and SDs), and LyveSETRel (*r*^2^ > 96.6 for LDs and SDs).

The Mantel test carried out with the B:NT:P1.5,2^∗^ LDs and SDs showed that the pipelines generally produced less similar genetic distances with the more closely related isolates, although the same large clusters were mostly retained (Supplementary Figures S2B, S3B). Thereby, SMALTPpl and kSNPRead pipelines generated mutually highly correlated output with all datasets (*r*^2^ > 97.2 with LDs and SDs). Lyveset-based pipelines, Parsnp and kSNPCon in some cases also co-clustered with SMALTPpl and kSNPRead but not for both all datasets (99.7 ≤ *r*^2^ ≤ 91.9), while cgMLST and PanseqSNP produced more distinct output (93.8 ≤ *r*^2^ ≤ 63.4). Results of a linear regression analysis largely confirmed the output of the Mantel test (Supplementary Figures S4, S5).

Additional tests were carried out comparing the output of Parsnp and SMALTPpl generated with the three different reference genomes between the references and to that of the other pipelines (Supplementary Figures S6, S7). With B:NT cc-269 isolate subsets (Supplementary Figures S6A, S7A), Parsnp produced highly correlated SNP distances with all three references (*r*^2^ > 98.6), while SMALTPpl showed somewhat larger differences between reference genomes, with hybrid Nanopore and NC017515.1 producing more correlated output to each other (*r*^2^ > 98.6) than to Pacbio (*r*^2^ > 95.7). Thereby, the output obtained using the Pacbio reference genome was more correlated to that of the other tested pipelines, followed by the hybrid Nanopore and NC017515.1 genome for SMALTPpl (*r*^2^ > 97.3 with Pacbio, *r*^2^ > 94.2 with hybrid Nanopore, and *r*^2^ > 92.7 with NC017515.1) and Parsnp pipelines (*r*^2^ > 96.9 with Pacbio, *r*^2^ > 96.5 with hybrid Nanopore and *r*^2^ > 95.2 with NC017515.1). With B:NT:P1.5,2^∗^, the output of SMALTPpl obtained using hybrid Nanopore and Pacbio was relatively similar between each other (*r*^2^ > 96.6), and to that of kSNPRead (*r*^2^ > 97.6), and highly different from that generated with NC017515.1 (*r*^2^ > 71.4) (Supplementary Figures S6B, S7B).

#### Phylogenetic Tree Comparison

**FIGURE 6:**
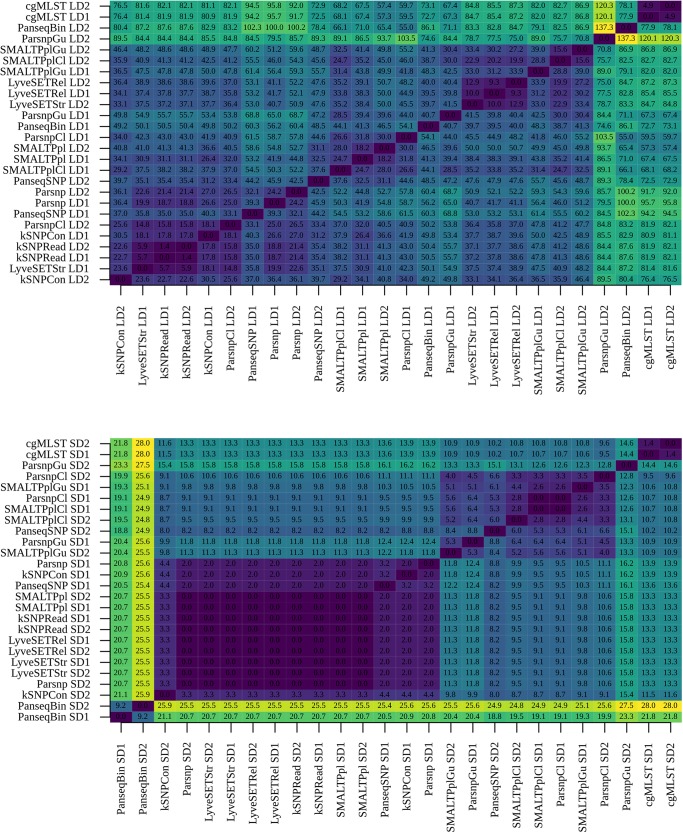
Comparison of phylogenetic trees between pipelines. Phylogenetic trees generated using B:NT cc-269 isolates from large datasets (LD1 and LD2) **(upper panel)** and small dataset (SD1 and SD2) **(lower panel)** were compared using Kendall–Colijn (KC) test for topology (λ = 0) and the obtained pairwise KC distances were reported.

With B:NT cc-269 SDs, SMALTPpl, LyveSET-based, kSNP-based Parsnp and PanseqSNP pipelines produced topologically nearly identical phylogenetic trees (Figure 6 lower panel). Also the trees made using either Gubbins or ClonalframeML showed relatively similar topology between each other, while PanseqBin and cgMLST trees were different from each other and from those made by other pipelines. The phylogenetic trees produced using cc-269 LDs displayed more variability (Figure 6 upper panel). Here, an intermediate similarity was observed between the trees generated by all pipelines, except cgMLST, and in some cases ParsnpGu, and PanseqBin which showed more different tree topologies. The main differences between the phylogenetic trees lay in the positions of B:NT:P1.14 ST269 isolates. cgMLST, which displayed distinct topology compared to other pipelines, grouped the majority of ST269 isolates to a separate clade, while in case of other pipelines they were distributed throughout the tree. Noticeably, the ParsnpGu pipeline included one B:NT:P1.14 ST269 isolate into the B:NT:P1.14 ST2693 clade with one of the datasets. Additional differences between the pipelines were observed within the two recurrent clades consisting of the B:NT:P1.5,2 ST269 isolates and the B:NT:P1.14 ST2693 isolates. kSNPRead and SMALTPpl were the only pipelines displaying identical arrangement of the B:NT:P1.5,2^∗^ isolates with all datasets and in case of SMALTPpl with all three reference genomes.

**FIGURE 7:**
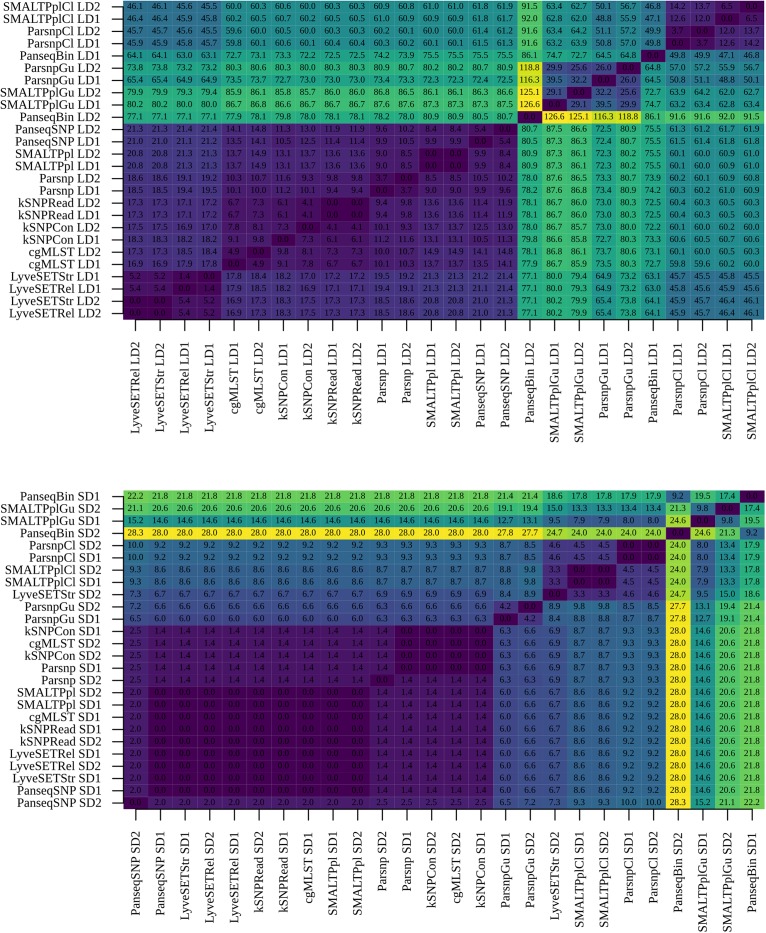
Comparison of UPGMA phylogenetic trees between pipelines. Phylogenetic trees of SNP-based subtyping workflows were generated using the UPGMA method and compared to the cgMLST and PanseqBin phylogenetic trees using Kendall–Colijn (KC) test for topology (λ = 0). The obtained pairwise KC distances were reported. The analysis was performed using B:NT cc-269 isolates from large datasets (LD1 and LD2) **(upper panel)** and small datasets (SD1 and SD2) **(lower panel)**.

Given the relatively linear genetic distances between some of the SNP-based pipelines and cgMLST, the low topological similarity between these workflows and cgMLST could result from the different tree-construction approaches: cgMLST trees were generated using the UPGMA method, while the trees of the SNP-based workflows were generated with maximum-likelihood method. To verify this, phylogenetic trees of all the SNP-based subtyping methods were re-created using UPGMA, and a new all-to-all comparison was carried out (Figure 7). The analysis showed that the UPGMA trees of PanseqSNP, kSNPCon, kSNPRead, SMALTPpl and Parsnp were indeed topologically similar to the cgMLST trees. The topology of the LyveSET trees was still more distinct from that observed for the listed SNP- and cgMLST-based workflows. Also the UPGMA trees created by workflows containing recombination-filtering tools were distinct from the cgMLST trees, as well as from the trees created by the SNP-based workflows without recombination filtering.

## Discussion

WGS is increasingly being applied in public health laboratories for surveillance and outbreak investigation of bacterial isolates. The use of WGS in routine and semi-routine requires the availability of data analysis tools that provide correct and consistent results for the organism in question, ensuring that the output is reliable and easy to interpret. However, no universal approaches exist for analysis of WGS data. Methods that are suitable to answer a particular research question need to be identified and tested separately for each organism or group of organisms with similar population genetics. In this study we have performed, as a proof of concept, a detailed comparison of different workflows for subtyping of *N. meningitidis*, including on the one hand investigation of the technical performance of the workflows, and on the other hand the functional comparison of the output between the methods.

The WGS-based analysis of the *N. meningitidis* isolate selection showed that B:NT isolates belonging to the cc-269, and three B:NT:P1.14 isolates with undetermined clonal complex formed a separate phylogenetic clade confirming the previous findings of Bertrand et al. (2011). Since the first description of the B:NT:P1.14 isolates (Bertrand et al., 2011), we have seen an increase in the prevalence of B:NT:P1.14 isolates belonging to ST2693. Moreover, current study showed that the B:NT:P1.14 ST2693 isolates were attributed to a separate branch within the cc-269 clade, while still being closely related to the B:NT:P1.14 ST269 cases. Besides, another cluster was detected within the B:NT cc-269 clade consisting of B:NT:P1.5,2 ST269 isolates harboring the VR1 5-1 allele for PorA. The B:NT:P1.5,2 ST269 isolates harboring the VR1 5 allele for PorA appeared not to be part of the latter cluster. Further, the serogroup W, Y and C isolates, were attributed to three separate isolate groups. However, the W and C isolates included in this study were always co-clustered, indicating that the serogroup W lineage might have emerged from the serogroup C lineage by a capsule switching event, which can be a subject of further investigation using available WGS data. Interestingly, the isolate B:NT:P1.14 belonging to cc-35 was stably found at the root of the B:NT:P1.14 cc-269 cluster indicating that they possibly emerged from a more recent common ancestor. The B:NT:P1.14 isolates belonging to cc-18, cc-162, and cc-213, on the contrary, were not closely related to the B:NT:P1.14 isolates belonging to cc-269, and to the other serogroup B, W, C, and Y isolates. Notably, the positions of the mentioned isolate groups were highly variable between workflows, and datasets. Possible factors that could have hampered stable reconstruction of the true phylogenetic relationship were the small number of isolates covering the phylogenetic branches of interest, and the lack of intermediate isolates to cover the large genetic distances between selected strains. The obtained results once more demonstrate the added value of WGS for outbreak investigation and surveillance, facilitating detection of disease clusters, their discrimination from the sporadic isolates, surveillance of known and emerging invasive strains, and description of the population structure of the pathogen of interest.

More detailed characterization of the workflows was performed using the set of B:NT isolates belonging to cc-269. Among the SNP-based workflows that do not use recombination filtering tools, all workflows produced mutually correlated and often linear SNP distances and similar phylogenetic trees. This could be sometimes influenced by the relatedness of the reference genome used as illustrated for the mapping-based pipeline SMALTPpl. Moreover, the genetic distances generated by cgMLST were relatively proportional to those generated by SNP-based pipelines. And while the cgMLST trees showed noticeable differences with the SNP-based trees, for instance attributing the B:NT:P1.14 ST269 isolates to a separate cluster within the B:NT cc-269 phylogenetic tree, additional tests illustrated that this was mostly caused by the differences in the phylogenetic tree construction methodologies, rather than the differences in the recorded genetic variation between isolates. In addition, both cgMLST and SNP-based workflows correctly grouped two large clades of closely related isolates to separate branches. Moreover, assessment of the technical performance characteristics indicated that Parsnp, PanseqSNP, kSNP-based workflows, SMALTPpl, and cgMLST showed stable performance with replicate datasets consisting of diverse isolates of the same cc, generating linear genetic distances and similar phylogenetic trees. The genetic distances returned by LyveSET-based workflows were more variable between the replicate datasets according to the linear regression analysis. Importantly, however, results of this study also suggested that the performance of cgMLST and PanseqSNP workflows, and to a lesser extent also of kSNPCon and Parsnp was less accurate for closely related isolates. This was demonstrated by a lower reproducibility and discriminatory power, inferior results of the Mantel test performed on the B:NT:P1.5,2 isolate subsets and, for cgMLST and PanseqSNP, inferior results of linear regression analysis on the same isolates. Among all of the tested pipelines, the best performance with closely related isolates was shown by SMALTPpl and kSNPRead, which showed good reproducibility and discriminatory power, correlated and linear SNP distances between each other and between replicate datasets for closely related isolates, and returned identical arrangement of these isolates between the replicate datasets on the phylogenetic trees. Finally, a small difference was observed in the coverage sensitivity of the workflows. The sequencing depth achieved in the low-coverage dataset by multiplexing 69 isolates on a single Miseq Illumina flow cell appeared to deliver sufficient coverage for subtyping with most of the tested workflows. The cgMLST pipeline, however, demonstrated a noticeable difference in the performance with the low-coverage dataset, compared to the one obtained with the high-coverage dataset, indicating that it could pose more stringent requirements for the input data. One of the tested methodologies, PanseqBin, appeared to be not suitable for subtyping of isolates according to most of the performed tests. Interestingly, this tool was still able to successfully group more closely related isolates in separate clades based on the analysis of the accessory genome only. We believe that this tool could be valuable for functional and comparative genomic analyses, bearing in mind its high sensitivity for the coverage of the input data. Moreover, the workflows which are showing lower performance with the current dataset may show better result with other datasets.

Our results demonstrated that cgMLST, as well as SNP-based approaches that use assembly, mapping and k-mer methodologies can show highly similar output for subtyping of isolates belonging to the same cc. Both cgMLST and SNP-based workflows can produce reliable genetic distances with replicate datasets, and the genetic distances can be highly correlated, and even linear, and thus interconvertible, between the workflows. This can be important in case that regulatory authorities rely on different approaches for subtyping of an organism and in case these data should be matched (e.g., clinical and food/environmental isolate in case of an outbreak) (Allard et al., 2016; Nadon et al., 2017). For the less closely related isolates, cgMLST can be applied directly, while for the SNP-based subtyping workflows, a more common strategy is to first subdivide isolates to groups with a sufficient level of relatedness to each-other, and if necessary to assign them to a reference genome, using classical subtyping information (Bårnes et al., 2017) or tools such as KmerID (Ashton et al., 2016). Given the advantages that are offered by the commonly used cgMLST scheme, i.e., the established nomenclature, the ease to store, compare and internationally exchange the results, and no need for a reference genome, we believe that it still represents the most optimal subtyping methodology for *N. meningitidis*. Our conclusions can, however, be important for organisms with a similar population structure for which no cgMLST schemes exist. Given the observed differences in the coverage sensitivity of cgMLST and SNP-based workflows, the latter can also be used for the analysis of input data of a lower coverage, potentially allowing to pool a larger number of isolates in a single sequencing run. Further, the obtained results suggest that for more closely related isolates, the accuracy of cgMLST and some of the SNP-based workflows might be not sufficient to guarantee reliable and meaningful output, which should be taken into account, e.g., when defining thresholds for outbreak delineation, or in case of an exhaustive outbreak investigation. Our observations show that workflows that use non-assembled read data as input such as kSNPReads and SMALTPpl might provide more accurate information for closely related isolates, although more extensive testing should be carried out with a larger number of cases to confirm this.

As elaborated above, among all of the tested pipelines, the best performance with closely related isolates was shown by SMALTPpl and kSNPRead. While kSNPRead is a reference-free approach, SMALTPpl requires a reference genome to run. The tool showed equally stable performance with all three reference genomes tested, but similarity of its output to that of the other pipelines was dependent on the relatedness level of the reference genome used. Moreover, the availability of a closely related reference genome is necessary if a more detailed characterization of isolates is envisaged, for instance in case of comparative genomic analysis. Generation of a closed reference genome requires the availability of long-read sequencing data, which currently can be obtained using two technologies: Pacbio and Nanopore. In this study, both approaches were tested, evaluating the quality of the obtained reference genome and its suitability for the high-resolution subtyping. Pacbio sequencing data generated using a single SMART cell appeared to be sufficient to create a closed high-quality reference using long-read data only. MinION sequencing data had a lower coverage, among others because two isolates were multiplexed on a single cell, and showed a higher error rate. A closed reference genome could still be obtained from MinION reads using hybrid assembly with Illumina sequencing data. The generated reference genome aligned along the entire length to the Pacbio reference, showing only a limited number of SNP differences and gaps. Despite the slightly lower quality of the MinION reference, the two reference genomes showed sufficiently similar performance characteristics, and facilitated calculation of linear SNP distance matrices and phylogenetic trees for both Parsnp and SMALTPpl, indicating that both of them are equally useful for subtyping and detailed characterization of *N. meningitidis* isolates. But compared to Pacbio, the Oxford Nanopore MinION sequencer is small, highly affordable and utilizes a relatively simple library preparation which makes the technology more accessible for the use in smaller public health laboratories. Besides, we showed that a reference genome of lower, but still sufficient quality can still be obtained if multiple genomes are sequenced on a single MinION flow cell if hybrid assembly with Illumina short reads is carried out, which could result in lower sequencing costs.

It has been shown previously that SNP-based subtyping approaches that do not use recombination filtering can generate phylogenetic trees that are similar to the real clonal genealogy (Hedge and Wilson, 2014; Didelot and Wilson, 2015) and thus potentially also to the topology obtained using cgMLST-based methods. Indeed, although recombination and other structural variations introduce multiple polymorphisms in a single evolutionary event, these polymorphisms still provide phylogenetically meaningful information that accumulates in the genome in the same time-dependent fashion as do mutations (Didelot and Wilson, 2015), allowing to reconstruct a topologically correct phylogeny. As discussed above, these observations are also supported by our results. However, it is currently widely assumed, and repeatedly demonstrated (Marttinen et al., 2012; Page et al., 2014; Didelot and Wilson, 2015), that the use of recombination filtering software allows to further improve the phylogenetic accuracy of SNP-based subtyping workflows and to correct the bias on the branch lengths that is introduced by recombination events. Therefore, another aim of this study was to evaluate the effect of recombination filtering tools on the performance and the output of SNP-based subtyping workflows. Interestingly, we showed that for *N. meningitidis*, the use of recombination filtering software resulted in more distinct genetic distances and phylogenetic tree topologies compared to the SNP-based workflows without recombination filtering and to cgMLST. Parsnp combined with Gubbins even resulted in some cases in a misplacement of isolates from otherwise stably recurring isolate clusters. These observations could be explained by the fact that (a) the SNP-based subtyping workflows and cgMLST do not reflect the correct clonal genealogy of *N. meningitidis*, (b) as *N. meningitidis* mainly evolves by recombination, there is a less well pronounced clonal geneaology, and filtering out recombination leaves insufficient signal for robust reconstruction of the population structure and (c) the recombination filtering tools did not perform correctly, because of not being fine-tuned to the high levels of recombination in the genome of *N. meningitidis*. In addition, pipelines using Gubbins showed an increased sensitivity to inter-run variation of the sequencing data compared to the pipelines in which recombination filtering step was omitted. For ClonalframeML, which performs a more relaxed SNP filtering, the output was less stable for the closely related isolates, showing a higher sensitivity for the inter-run variation and coverage of the input data, and reference genome compared to the original workflow. Notably, among the tested pipelines, the most similar output to the ClonalframeML and Gubbins workflows was demonstrated by the LyveSET-based workflows. The LyveSET pipeline allows to perform extensive SNP quality filtering, including a SNP pruning step, i.e., removal of SNPs that are located too closely to each other on the chromosome. SNP pruning is used to mask polymorphisms from regions that show a too high SNP frequency, discarding among others a fraction of polymorphisms arising from recombination events, which could explain the observed similarities to ClonalframeML. Taken together, our observations suggest that caution should be associated with using a recombination filtering step or extensive SNP pruning for subtyping of *N. meningitidis.* However, both of the tested recombination filtering tools, and the LyveSET pipeline are undoubtfully useful for finding recombinant regions in case of comparative genomic studies of *N. meningitidis*, or for subtyping of species with a different, more clonal population structure. Besides, the applied tools could possibly be fine-tuned for a more accurate performance with *N. meningitidis*.

Current work focuses on the evaluation of WGS-based workflows using different approaches. The established methodology, relying on the calculation of performance metrics, permits to relatively quickly describe the different characteristics of the workflows. A newer approach, proposed by Katz et al. (2017) and applied in this study, can be used for a more detailed evaluation of pipelines. While this strategy is more computationally demanding, it allows to carry out a detailed comparison of subtyping workflows between each-other, and in case that multiple testing datasets are available, to select methods that show the most stable performance with a limited hands-on time. The differences that were observed between the subtyping workflows in this study demonstrate that there is a need to continue to benchmark data analysis pipelines, ensuring that the applied methodologies are suitable for the species and research question of interest, and that information extracted from the data remains exchangeable between international public health laboratories. Moreover, in the future, a set of requirements should be specified for the workflows to be considered as valid. Hereto initiatives such as GMI where a set of benchmarking datasets and eventually also metrics for pipeline comparisons are being made available (Timme et al., 2017), will contribute considerably to the standardization and harmonization of the data analysis tools used in public health laboratories.

## Data Availability Statement

The datasets generated for this study can be found in the NCBI Sequence Read Archive under Accession Number PRJNA549235.

## Author Contributions

AS, KM, and SD conceived and designed this study. SD supervised the project. AS performed all bioinformatics analysis. WM and SB collected and isolated DNA of *N*. *meningitidis* samples to be used in this study, and provided specialist feedback on the obtained results on *Neisseria* relatedness. AS, NR, KM, SB, and SD participated in the interpretation of the results. AS and SD wrote the draft of the manuscript. All authors read and approved the submitted version.

## Conflict of Interest

The authors declare that the research was conducted in the absence of any commercial or financial relationships that could be construed as a potential conflict of interest.

## Footnotes

1 http://pubmlst.org/

2 https://github.com/rrwick/Porechop

3 https://github.com/phe-bioinformatics/kmerid

4 https://github.com/kwongj/maskrc-svg

5 https://github.com/lskatz/Lyve-SET-paper/blob/master/compareSnps.sh

6 https://github.com/lskatz/Lyve-SET-paper/blob/master/compareTrees.sh

7 https://github.com/thibautjombart/treespace
