# Supplementary material for: Detailed Evaluation of Data Analysis Tools for Subtyping of Bacterial Isolates Based on Whole Genome Sequencing: Neisseria meningitidis as a Proof of Concept
Source: Front Microbiol. 2019 Dec 18;10:2897. doi: 10.3389/fmicb.2019.02897 (PMC6930190; doi:10.3389/fmicb.2019.02897)
Supplement: Supplementary file 1 [file Data_Sheet_1.docx]

Supplementary Material

# Supplementary Data

**Data Sheet S1: Phylogenetic trees can be downloaded from https://doi.org/10.5281/zenodo.3248936.**

trees_all_isolates.zip: phylogenetic trees generated using all isolates

trees_BNT_cc269_isolates.zip: phylogenetic trees generated using cc-269 isolates

trees_upgma_BNT_cc269_isolates.zip: phylogenetic trees generated using cc-269 isolates and UPGMA tree construction methodology

**Common legend:** Color codes: **red**: serogroup W, **yellow**: serogroup Y, **purple**: serogroup C, **green**: serogroup B isolates belonging to cc-269 clonal complex except one sub-branch, B:NT:P1.5,2*, which is colored **olive**, **cyan**: serogroup B isolates belonging to other clonal complexes than cc-269, **black**: serogroup B isolates with undetermined clonal complex. Two clades of cc-269 isolates discussed in more detail in the main text, B:NT:P1.14 ST2693 and B:NT:P1.5,2* are indicated in **bold**. ND: not determined, STND: sequence type not determined, NE: clonal complex does not exist.

# Supplementary Figures and Tables

## Supplementary Figures


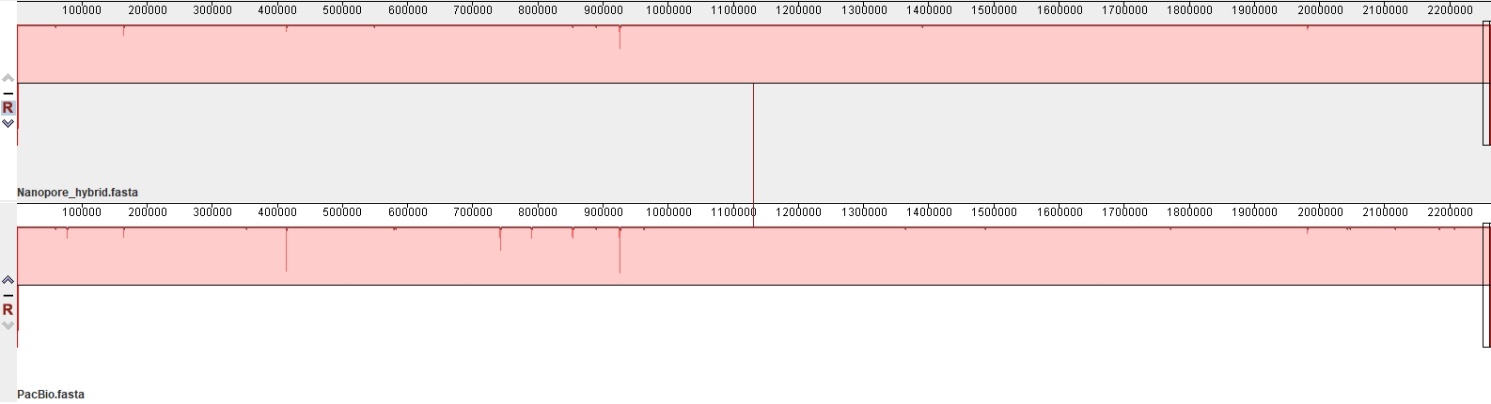


**Figure S1. Reference genomes.** Alignment between the hybrid Nanopore (above) and long-read-only PacBio (below) genome assemblies generated using progressive Mauve aligner (Darling et al., 2010). The two assemblies align along the entire length, showing only 24 SNPs and 51 gaps with a maximal size of 52 bp, represented by short vertical lines.

**
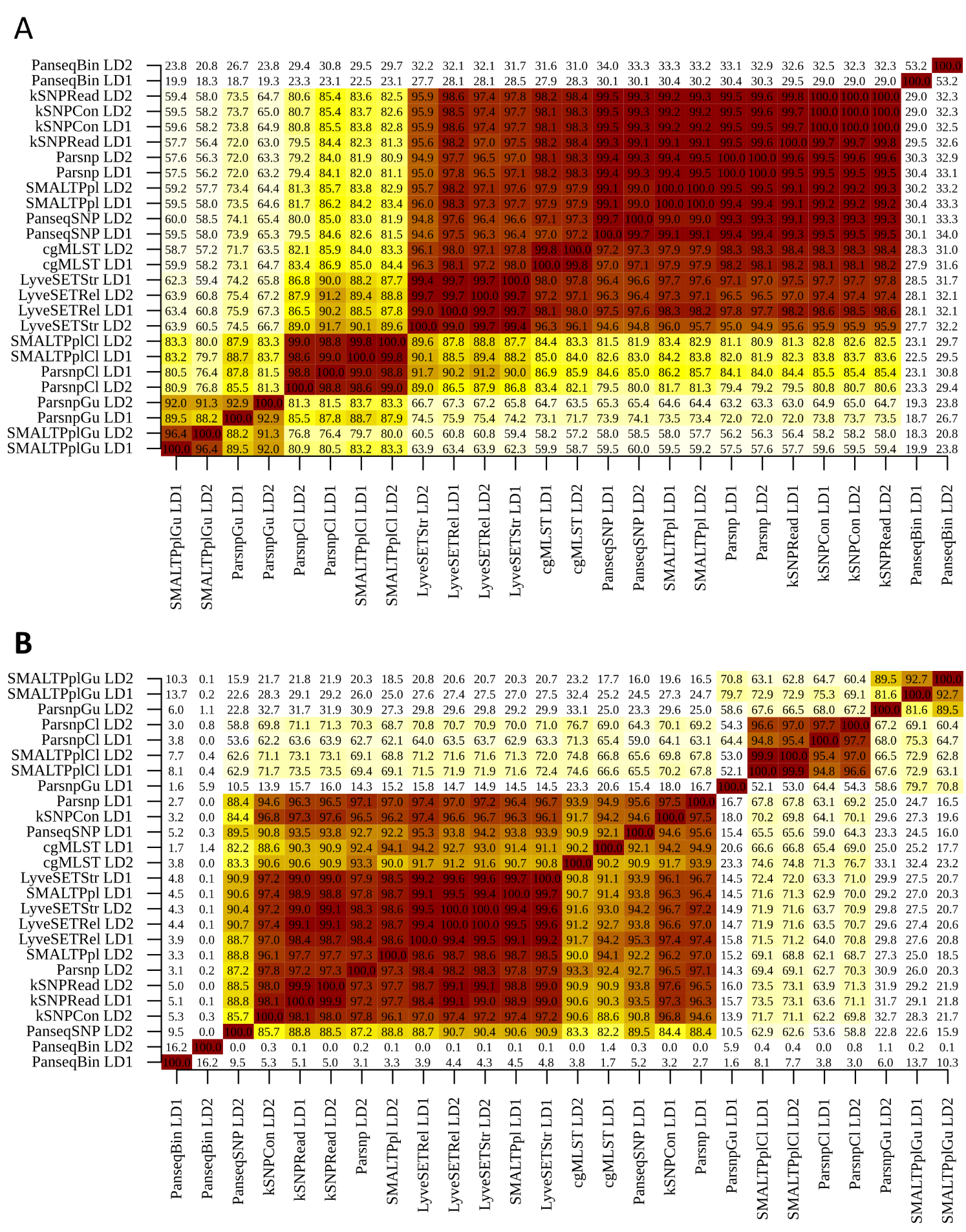
**

**Figure S2. Comparison of genetic distance matrices between pipelines with Mantel test – large datasets.** Distance matrices generated with B:NT cc-269 isolates **(upper panel)** and the more closely related B:NT:P1.5,2* isolates **(lower panel)** from large datasets (LD1 and LD2) were compared using Mantel test, and the obtained Spearman correlation coefficients (r^2^) were reported.

**
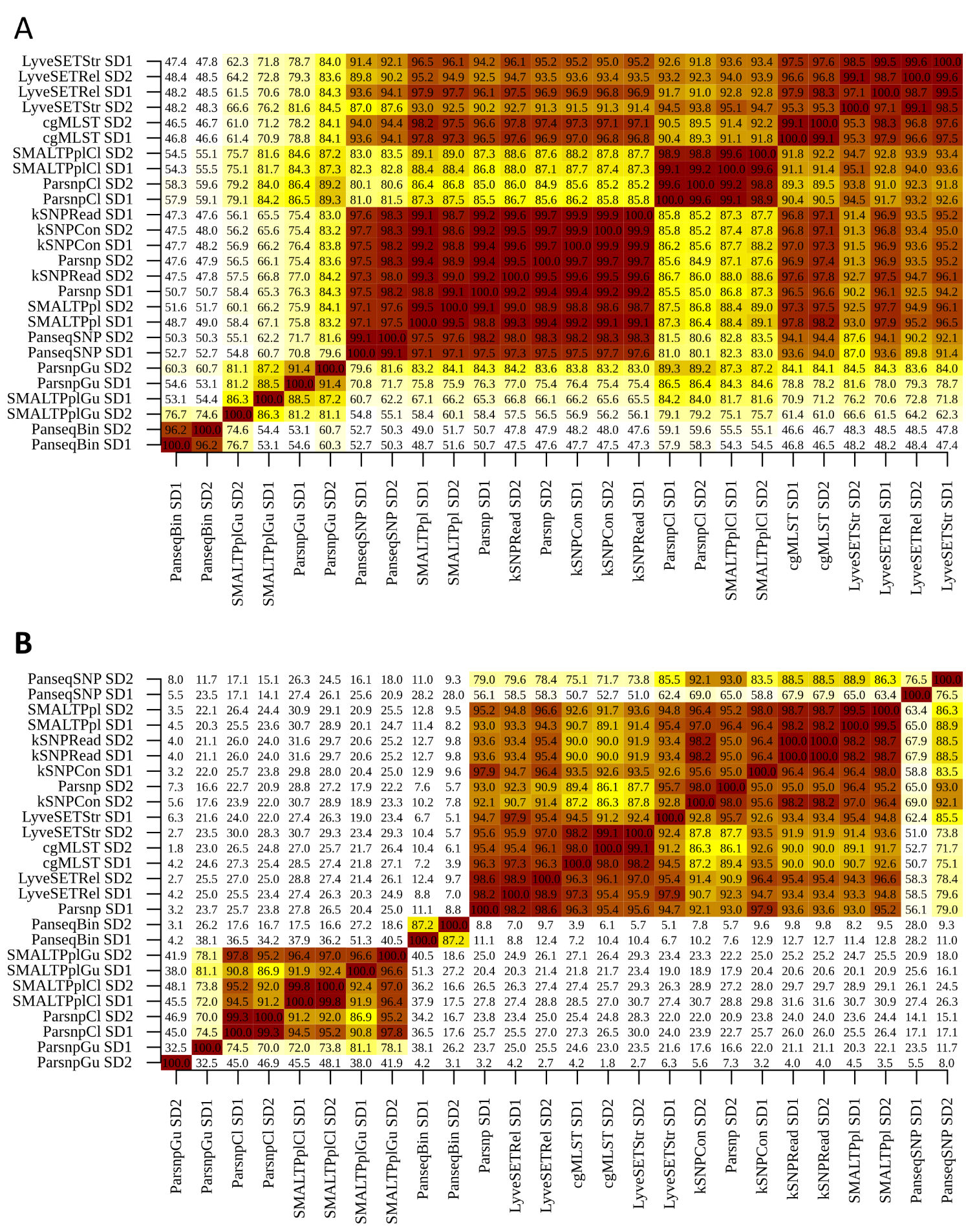
 Figure S3. Comparison of genetic distance matrices between pipelines with Mantel test – small datasets.** Distance matrices generated with B:NT cc-269 isolates **(A)** and the more closely related B:NT:P1.5,2* isolates **(B)** from small datasets (SD1 and SD2) were compared using Mantel test, and the obtained Spearman correlation coefficients (r^2^) were reported.

**
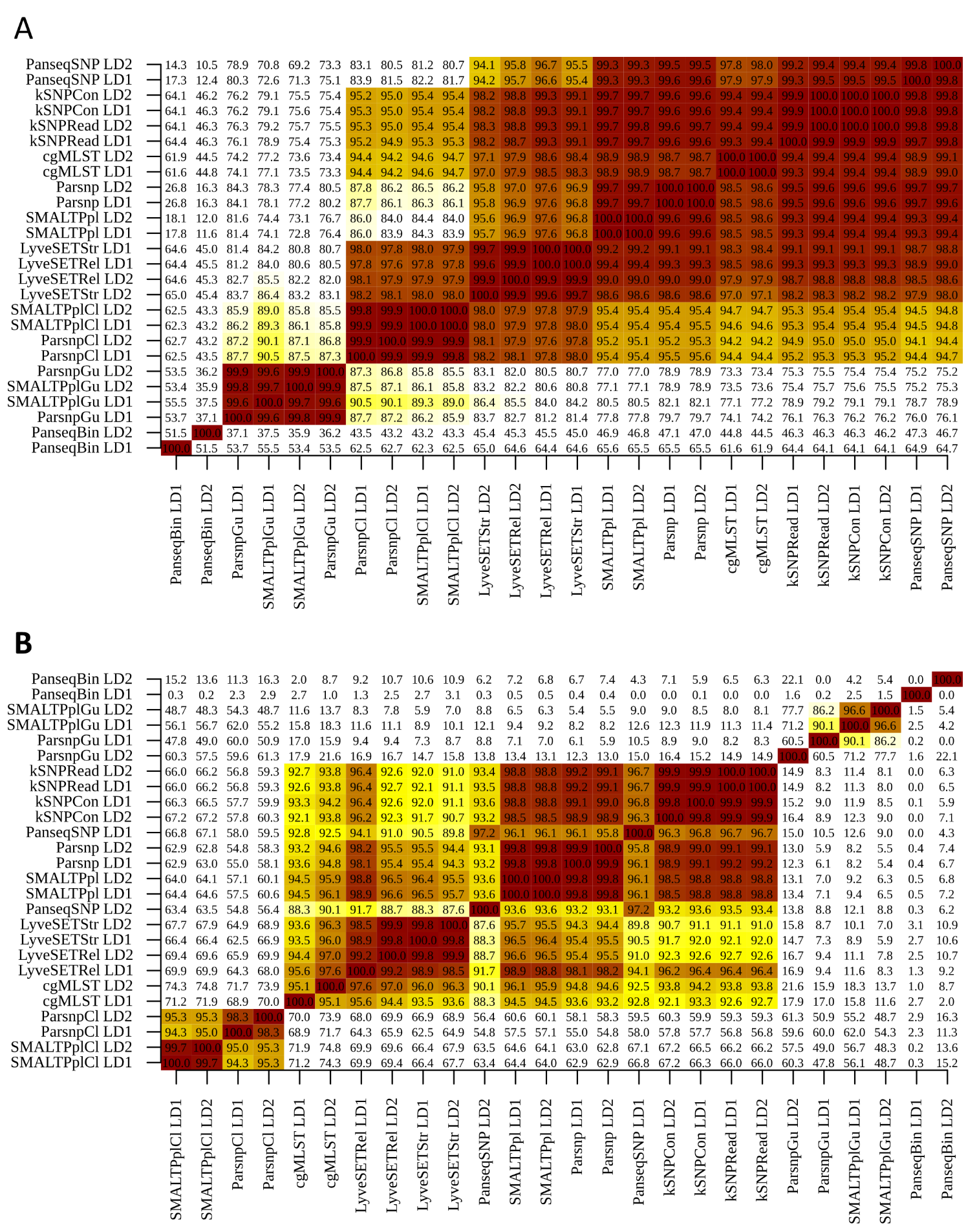
 Figure S4. Comparison of genetic distance matrices between pipelines by means of linear regression – large datasets.** Linear regression analysis was performed between distance matrices generated using B:NT cc-269 isolates **(A)** and the more closely related B:NT:P1.5,2* isolates **(B)** from large dataset (LD1 and LD2), and the obtained correlation coefficients (r^2^) were reported.

**
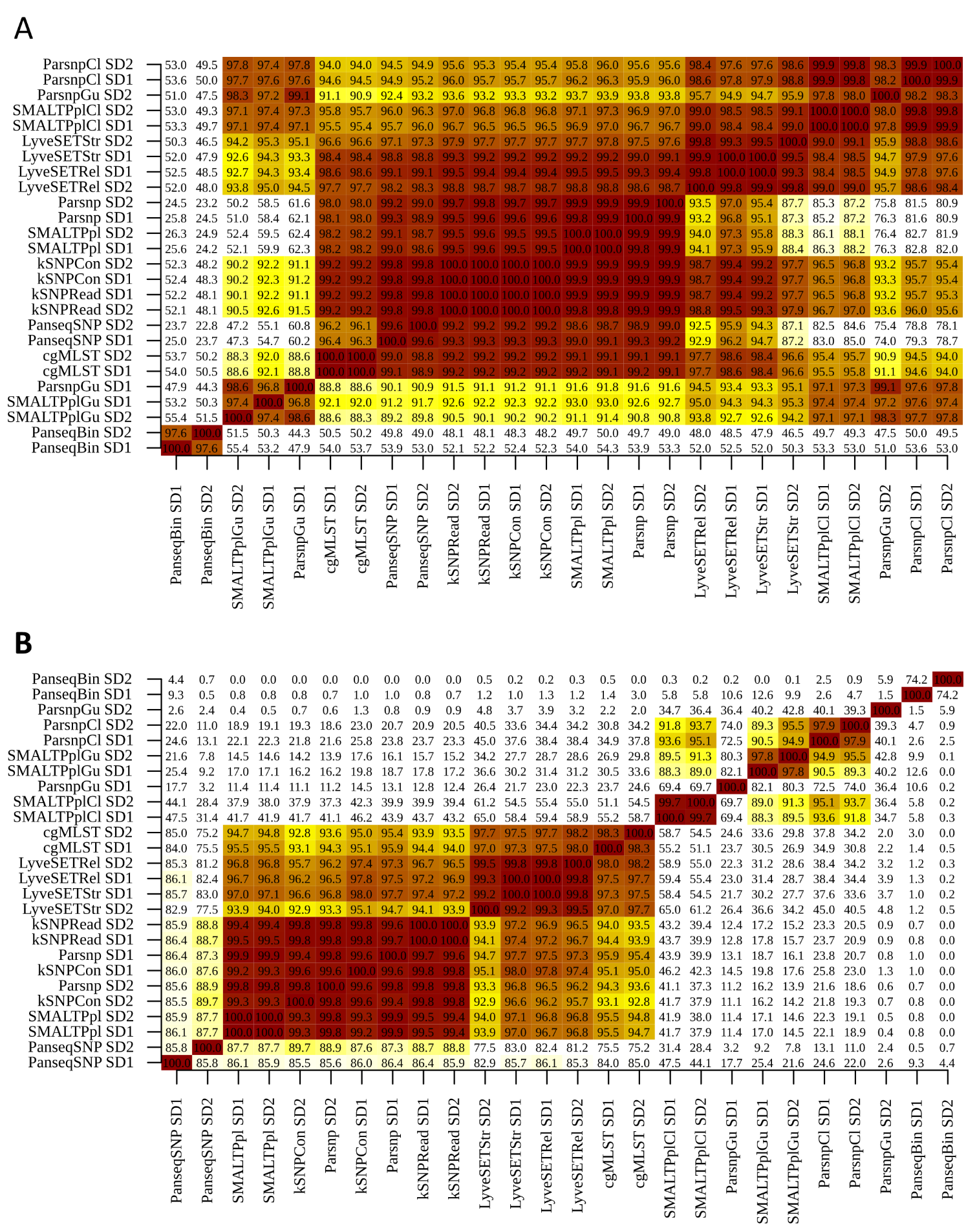
 Figure S5. Comparison of genetic distance matrices between pipelines by means of linear regression – small datasets.** Linear regression analysis was performed between distance matrices generated using B:NT cc-269 isolates **(A)** and the more closely related B:NT:P1.5,2* isolates **(B)** from small dataset (SD1 and SD2), and the obtained correlation coefficients (r^2^) were reported.

**
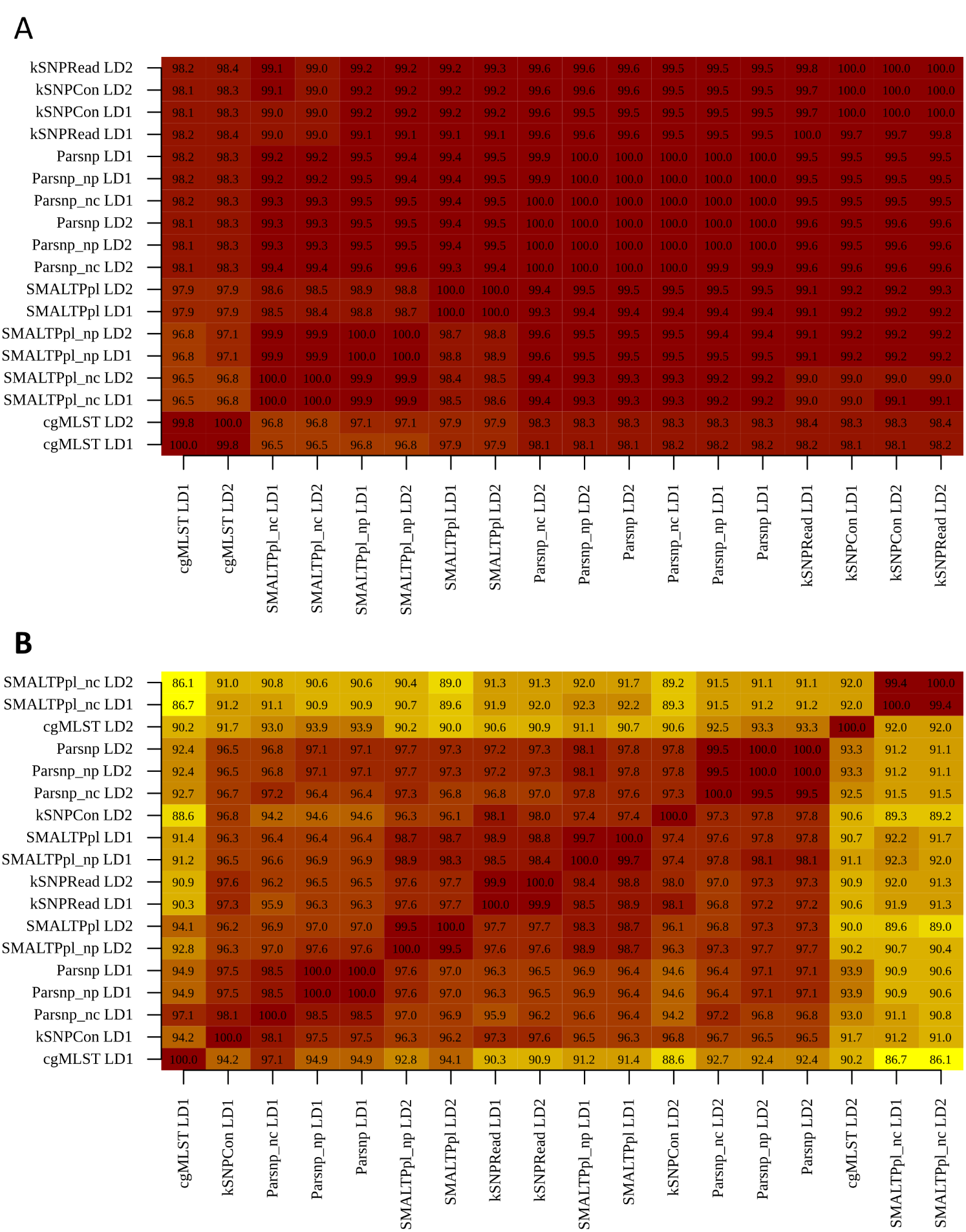
 Figure S6. Comparison of genetic distance matrices between selected pipelines applied with different reference genomes by means of the Mantel test – large datasets.** Linear regression analysis was performed between distance matrices generated using B:NT cc-269 isolates **(A)** and the more closely related B:NT:P1.5,2* isolates **(B)** from large dataset (LD1 and LD2), and the obtained correlation coefficients (r^2^) were reported. SMALTPpl and Parsnp were applied with three different reference genomes, more specifically Pacbio (/), hybrid Nanopore (np) and NC017515.1 (nc).

**
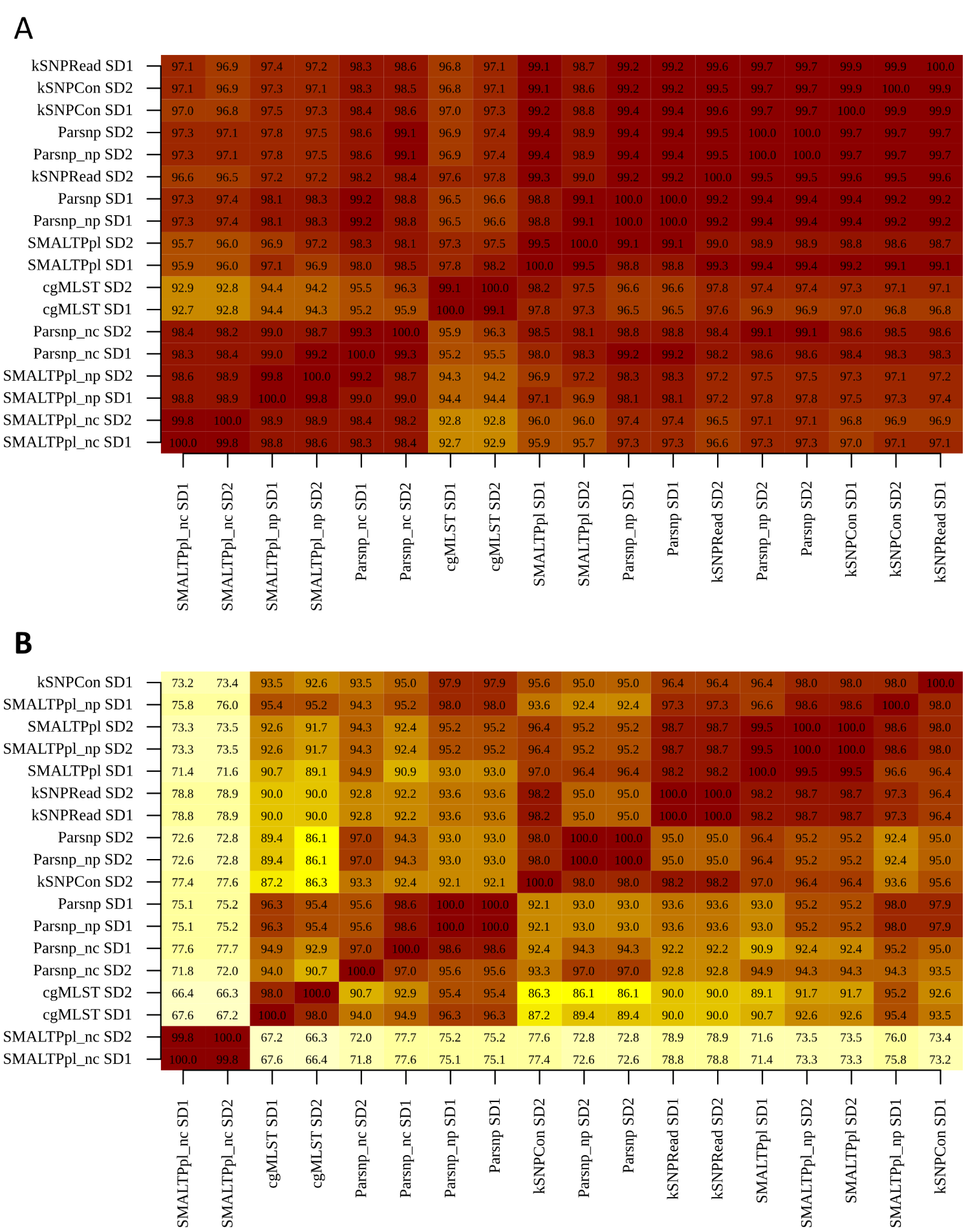
 Figure S7. Comparison of genetic distance matrices between selected pipelines applied with different reference genomes by means of the Mantel test – small datasets.** Linear regression analysis was performed between distance matrices generated using B:NT cc-269 isolates **(A)** and the more closely related B:NT:P1.5,2* isolates **(B)** from small dataset (SD1 and SD2), and the obtained correlation coefficients (r^2^) were reported. SMALTPpl and Parsnp were applied with three different reference genomes, more specifically Pacbio (/), hybrid Nanopore (np) and NC017515.1 (nc).

## Supplementary Tables

**Table S1. Number of reads and sequencing coverage of isolates in the four sequencing datasets.** Coverage was determined using Qualimap 2.2.1 (Okonechnikov et al., 2016) based on reads mapped on PacBio reference genome (see Materials and Methods) with SMALT 0.7.6 (Ponstingl and Ning, 2010) at default parameters.

|  | Large dataset 1 | | Large dataset 2 | | Small dataset 1 | | Small dataset 2 | |
| --- | --- | --- | --- | --- | --- | --- | --- | --- |
| Sample name | Number of reads | Coverage mean | Number of reads | Coverage mean | Number of reads | Coverage mean | Number of reads | Coverage mean |
| 2002-116 | 428513 | 72 | 375942 | 64 |  |  |  |  |
| 2003-047 | 229669 | 53 | 453987 | 98 |  |  |  |  |
| 2004-065 | 386104 | 63 | 363368 | 60 |  |  |  |  |
| 2005-190 | 312688 | 72 | 292336 | 66 |  |  |  |  |
| 2006-171 | 459673 | 97 | 445423 | 93 |  |  |  |  |
| 2007-018 | 379527 | 88 | 358518 | 82 |  |  |  |  |
| 2007-051 | 416002 | 73 | 356708 | 63 |  |  |  |  |
| 2007-080 | 339682 | 82 | 330233 | 79 |  |  |  |  |
| 2007-172 | 321907 | 70 | 320402 | 69 |  |  |  |  |
| 2008-034 | 309616 | 47 | 293434 | 45 |  |  |  |  |
| 2008-060 | 370894 | 71 | 337615 | 64 |  |  |  |  |
| 2008-114 | 435154 | 79 | 414681 | 75 |  |  |  |  |
| 2008-120 | 303212 | 71 | 302208 | 71 |  |  |  |  |
| 2009-014 | 423884 | 85 | 415025 | 83 |  |  |  |  |
| 2009-020 | 426758 | 68 | 419192 | 67 |  |  |  |  |
| 2009-098 | 398925 | 82 | 396576 | 81 |  |  |  |  |
| 2009-105 | 477168 | 80 | 458327 | 77 |  |  |  |  |
| 2010-129 | 481708 | 95 | 469622 | 92 |  |  |  |  |
| 2011-004 | 366314 | 72 | 349838 | 69 |  |  |  |  |
| 2011-005 | 486827 | 78 | 465288 | 75 |  |  |  |  |
| 2011-006 | 370979 | 75 | 354119 | 71 | 500606 | 115 | 273321 | 65 |
| 2011-010 | 427717 | 79 | 371380 | 69 | 593572 | 137 | 300163 | 71 |
| 2011-023 | 478965 | 80 | 437871 | 74 |  |  |  |  |
| 2011-025 | 246129 | 56 | 237217 | 54 | 566721 | 139 | 273183 | 68 |
| 2011-027 | 397174 | 81 | 368717 | 75 | 455343 | 111 | 255967 | 63 |
| 2011-042 | 296653 | 71 | 290835 | 69 |  |  |  |  |
| 2011-058 | 460288 | 87 | 438002 | 83 |  |  |  |  |
| 2011-086 | 328409 | 81 | 328024 | 80 | 893783 | 217 | 396327 | 98 |
| 2012-079 | 475824 | 88 | 449518 | 83 |  |  |  |  |
| S13BD01093 | 255210 | 57 | 393638 | 88 |  |  |  |  |
| S13BD02289 | 274444 | 67 | 261756 | 63 |  |  |  |  |
| S15BD00757 | 671853 | 145 | 662029 | 142 |  |  |  |  |
| S15BD01319 | 389026 | 89 | 355690 | 81 | 832285 | 191 | 583678 | 139 |
| S15BD03615 | 375560 | 64 | 354519 | 61 | 579760 | 139 | 856224 | 210 |
| S13BD00117 | 502895 | 93 | 478615 | 89 | 1729604 | 400 | 437229 | 106 |
| S13BD00431 | 499548 | 97 | 485778 | 94 | 1480457 | 336 | 987230 | 235 |
| S13BD00761 | 195456 | 43 | 540495 | 120 |  |  |  |  |
| S13BD01533 | 183608 | 44 | 1480998 | 302 | 879474 | 206 | 1027167 | 249 |
| S13BD01748 | 324373 | 64 | 306509 | 60 |  |  |  |  |
| S13BD02841 | 246971 | 58 | 321729 | 81 |  |  |  |  |
| S13BD03579 | 421371 | 93 | 404513 | 89 |  |  |  |  |
| S13BD03733 | 474229 | 77 | 444178 | 72 |  |  |  |  |
| S13BD03907 | 383304 | 74 | 359621 | 69 | 1023185 | 225 | 905693 | 205 |
| S14BD01180 | 399478 | 89 | 390281 | 86 |  |  |  |  |
| S14BD01880 | 482039 | 92 | 459771 | 88 | 450770 | 110 | 440745 | 109 |
| S14BD04646 | 341797 | 61 | 339639 | 61 | 570014 | 130 | 328485 | 76 |
| S15BD00088 | 469910 | 75 | 424714 | 69 |  |  |  |  |
| S15BD05018 | 117111 | 22 | 402038 | 100 |  |  |  |  |
| S15BD07026 | 246457 | 53 | 599574 | 131 |  |  |  |  |
| S15BD00217 | 417945 | 68 | 405372 | 67 | 797915 | 176 | 609788 | 138 |
| S15BD02364 | 556850 | 103 | 486712 | 91 |  |  |  |  |
| S15BD06042 | 477089 | 76 | 449223 | 73 | 554543 | 135 | 468716 | 116 |
| 2009-015 | 436451 | 81 | 432757 | 80 |  |  |  |  |
| 2012-152 | 440185 | 73 | 410102 | 68 | 632466 | 153 | 492157 | 121 |
| S13BD03226 | 392898 | 88 | 387758 | 86 | 973484 | 224 | 702676 | 165 |
| S14BD04865 | 377048 | 69 | 367521 | 67 | 976620 | 233 | 778323 | 192 |
| S15BD01379 | 369175 | 67 | 358183 | 65 |  |  |  |  |
| S15BD04089 | 252132 | 58 | 242586 | 55 | 1175265 | 262 | 1086290 | 250 |
| S13BD03199 | 542248 | 93 | 529693 | 92 |  |  |  |  |
| S13BD03739 | 542648 | 88 | 520077 | 85 | 954656 | 216 | 769265 | 178 |
| S15BD02596 | 613500 | 110 | 604238 | 109 |  |  |  |  |
| S15BD09234 | 410912 | 80 | 372831 | 72 |  |  |  |  |
| S16BD01507 | 533196 | 80 | 511154 | 77 |  |  |  |  |
| S16BD01540 | 359330 | 74 | 353289 | 72 | 1385144 | 304 | 619847 | 141 |
| 2012-040 | 365055 | 81 | 363358 | 80 | 1020449 | 235 | 543550 | 129 |
| S13BD01417 | 230049 | 53 | 274208 | 62 | 1735212 | 374 | 904542 | 205 |
| S14BD01395 | 280181 | 64 | 275853 | 62 | 1577474 | 341 | 752955 | 170 |
| S14BD01857 | 322360 | 71 | 320727 | 71 |  |  |  |  |
| S15BD05503 | 420936 | 86 | 407611 | 83 |  |  |  |  |
| Mean: | 388858 | 76 | 412083 | 81 | 930783 | 213 | 616397 | 146 |

**Table S2. Performance metrics of the workflows.** Performance metrics of the workflows were determined with the B:NT cc-269 isolate subsets from the two large datasets, B:NT cc-269 LDs **(A)**, the two small datasets, B:NT cc-269 SDs **(B)**, and the two composite small datasets B:NT cc-269 cSDs **(C)**. The table shows the discriminatory power (D), number of observed subtypes (subtypes), threshold used for the calculation of discriminatory power (TH), which corresponds to the maximal number of SNPs detected between replicate isolates, and the size of the genetic matrix (matrix size) calculated for the listed pipelines using different datasets.

|  | **A. B:NT cc-269 LDs** | | | | | **B. B:NT cc-269 SDs** | | | | | **C. B:NT cc-269 cSDs** | | | | |
| --- | --- | --- | --- | --- | --- | --- | --- | --- | --- | --- | --- | --- | --- | --- | --- |
| **Pipeline** | **Data set** | **# sub types** | **D** | **TH** | **Matrix size** | **Data set** | **# sub types** | **D** | **TH** | **Matrix size** | **Data set** | **# sub types** | **D** | **TH** | **Matrix size** |
| cgMLST | LD1 | 31 | 0.984 | 9 | 1086 | SD1 | 13 | 0.989 | 4 | 654 | cSD1 | 12 | 0.978 | 9 | 654 |
| cgMLST | LD2 | 30 | 0.979 | 10 | 1083 | SD2 | 13 | 0.989 | 4 | 652 | cSD2 | 10 | 0.923 | 12 | 653 |
| PanseqBin | LD1 | 29 | 0.970 | 182 | 3956 | SD1 | 6 | 0.769 | 87 | 525 | cSD1 | 12 | 0.967 | 204 | 1933 |
| PanseqBin | LD2 | 26 | 0.952 | 186 | 3603 | SD2 | 7 | 0.857 | 77 | 525 | cSD2 | 11 | 0.956 | 203 | 1956 |
| PanseqSNP | LD1 | 34 | 0.993 | 140 | 35665 | SD1 | 12 | 0.978 | 273 | 19766 | cSD1 | 13 | 0.989 | 182 | 19297 |
| PanseqSNP | LD2 | 35 | 0.996 | 130 | 35880 | SD2 | 13 | 0.989 | 252 | 19614 | cSD2 | 12 | 0.978 | 144 | 19249 |
| Parsnp | LD1 | 33 | 0.989 | 11 | 33808 | SD1 | 11 | 0.956 | 23 | 17676 | cSD1 | 13 | 0.989 | 14 | 17755 |
| Parsnp | LD2 | 36 | 0.996 | 10 | 33814 | SD2 | 11 | 0.956 | 24 | 17668 | cSD2 | 13 | 0.989 | 12 | 17774 |
| kSNPCon | LD1 | 34 | 0.993 | 12 | 10415 | SD1 | 12 | 0.978 | 12 | 5316 | cSD1 | 13 | 0.989 | 12 | 5300 |
| kSNPCon | LD2 | 36 | 0.997 | 12 | 10419 | SD2 | 14 | 1.000 | 12 | 5308 | cSD2 | 14 | 1.000 | 12 | 5314 |
| kSNPRead | LD1 | 38 | 1.000 | 0 | 10196 | SD1 | 14 | 1.000 | 0 | 5247 | cSD1 | 14 | 1.000 | 0 | 5243 |
| kSNPRead | LD2 | 38 | 1.000 | 0 | 10288 | SD2 | 14 | 1.000 | 0 | 5004 | cSD2 | 14 | 1.000 | 0 | 5256 |
| SMALTPpl | LD1 | 37 | 0.999 | 1 | 34950 | SD1 | 13 | 0.989 | 4 | 18430 | cSD1 | 13 | 0.989 | 1 | 18217 |
| SMALTPpl | LD2 | 37 | 0.999 | 1 | 34893 | SD2 | 13 | 0.989 | 4 | 18103 | cSD2 | 13 | 0.989 | 1 | 18145 |
| LyveSETRel | LD1 | 38 | 1.000 | 0 | 6361 | SD1 | 14 | 1.000 | 0 | 3422 | cSD1 | 14 | 1.000 | 0 | 3122 |
| LyveSETRel | LD2 | 38 | 1.000 | 0 | 5989 | SD2 | 14 | 1.000 | 0 | 3136 | cSD2 | 14 | 1.000 | 0 | 2910 |
| LyveSETStr | LD1 | 38 | 1.000 | 0 | 5793 | SD1 | 14 | 1.000 | 0 | 3166 | cSD1 | 14 | 1.000 | 0 | 2821 |
| LyveSETStr | LD2 | 38 | 1.000 | 0 | 5178 | SD2 | 14 | 1.000 | 0 | 2665 | cSD2 | 14 | 1.000 | 0 | 2506 |
| **Pipelines with recombination filtering** | | | | | | | | | | | | | | | |
| ParsnpCl | LD1 | 37 | 0.999 | 1 | 1669 | SD1 | 11 | 0.956 | 5 | 641 | cSD1 | 14 | 1.000 | 3 | 656 |
| ParsnpCl | LD2 | 37 | 0.999 | 1 | 1652 | SD2 | 13 | 0.989 | 5 | 642 | cSD2 | 14 | 1.000 | 4 | 678 |
| ParsnpGu | LD1 | 27 | 0.976 | 8 | 944 | SD1 | 13 | 0.989 | 8 | 320 | cSD1 | 11 | 0.956 | 11 | 299 |
| ParsnpGu | LD2 | 32 | 0.987 | 5 | 929 | SD2 | 14 | 1.000 | 7 | 292 | cSD2 | 13 | 0.989 | 9 | 307 |
| SMALTPplCl | LD1 | 37 | 0.999 | 1 | 1726 | SD1 | 14 | 1.000 | 0 | 657 | cSD1 | 13 | 0.989 | 1 | 661 |
| SMALTPplCl | LD2 | 37 | 0.999 | 1 | 1723 | SD2 | 14 | 1.000 | 0 | 650 | cSD2 | 13 | 0.989 | 1 | 664 |
| SMALTPplGu | LD1 | 38 | 1.000 | 0 | 886 | SD1 | 12 | 0.967 | 4 | 287 | cSD1 | 14 | 1.000 | 0 | 270 |
| SMALTPplGu | LD2 | 38 | 1.000 | 0 | 978 | SD2 | 12 | 0.967 | 4 | 324 | cSD2 | 14 | 1.000 | 0 | 300 |

**Table S3. Stability of the tested workflows towards inter-run variability: Mantel test.** The table displays output of Mantel test accessing Spearman’s rank-order correlation (r^2^) to compare pairwise SNP distances generated by the different workflows using replicate sequencing datasets. The analysis was performed for the B:NT cc-269, and the more closely related B:NT:P1.5,2* isolate subsets of two large datasets, LDs **(A)**, two small datasets, SDs **(B)** and two composite small datasets, cSDs **(C)**.

|  | **B:NT cc-269 isolates** | | | **B:NT:P1.5,2* isolates** | | |
| --- | --- | --- | --- | --- | --- | --- |
| **Pipeline** | **A. LDs** | **B. SDs** | **C. cSDs** | **A. LDs** | **B. SDs** | **C. cSDs** |
| cgMLST | 99.8 | 99.1 | 99.2 | 90.2 | 98.0 | 70.4 |
| PanseqBin | 53.2 | 96.2 | 24.3 | 16.2 | 87.2 | 24.0 |
| PanseqSNP | 99.7 | 99.1 | 99.3 | 89.5 | 76.5 | 83.5 |
| Parsnp | 100.0 | 99.4 | 99.6 | 97.1 | 93.0 | 95.7 |
| kSNPCon | 100.0 | 99.9 | 99.8 | 96.8 | 95.6 | 91.0 |
| kSNPRead | 99.8 | 99.6 | 99.9 | 99.9 | 100.0 | 99.3 |
| SMALTPpl | 100.0 | 99.5 | 99.7 | 98.7 | 99.5 | 99.6 |
| LyveSETRel | 99.7 | 98.7 | 98.6 | 99.4 | 98.9 | 98.9 |
| LyveSETStr | 99.4 | 98.5 | 99.3 | 99.6 | 92.4 | 98.7 |
| **Pipelines with recombination filtering** | | | | | | |
| ParsnpCl | 98.8 | 99.6 | 99.1 | 97.7 | 99.3 | 86.6 |
| ParsnpGu | 92.9 | 91.4 | 90.2 | 58.6 | 32.5 | 59.4 |
| SMALTPplCl | 99.8 | 99.6 | 99.2 | 99.9 | 99.8 | 90.3 |
| SMALTPplGu | 96.4 | 86.3 | 96.8 | 92.7 | 96.6 | 100.0 |

**Table S4. stability of the tested workflows towards inter-run variability: linear regression analysis.** The table displays output of linear regression analysis (equation, r^2^) comparing pairwise SNP distances generated by the different workflows using replicate sequencing datasets. The analysis was performed for the B:NT cc-269, and the more closely related B:NT:P1.5,2* isolate subsets of two large datasets, LDs **(A)**, two small datasets, SDs **(B)** and two composite small datasets, cSDs **(C)**.

|  | **B:NT cc-269 isolates** | | | | | | **B:NT:P1.5,2* isolates** | | | | | |
| --- | --- | --- | --- | --- | --- | --- | --- | --- | --- | --- | --- | --- |
|  | **A. LDs** | | **B. SDs** | | **C. cSDs** | | **A. LDs** | | **B. SDs** | | **C. cSDs** | |
| **Pipeline** | **equation** | **r^2^** | **equation** | **r^2^** | **equation** | **r^2^** | **equation** | **r^2^** | **equation** | **r^2^** | **equation** | **r^2^** |
| cgMLST | y=1.001x+-0.5733 | 100.0 | y=1.002x+0.2394 | 100.0 | y=0.9945x+0.4172 | 99.9 | y=0.9981x+0.07672 | 95.1 | y=0.9123x+1.11 | 98.3 | y=0.9789x+-0.08833 | 88.4 |
| PanseqBin | y=0.7304x+58.16 | 51.5 | y=0.9848x+1.888 | 97.6 | y=0.4593x+179.9 | 17.0 | y=0.01681x+228.8 | 0.0 | y=1.157x+-4.51 | 74.2 | y=-0.947x+492.4 | 14.5 |
| PanseqSNP | y=1.002x+47.99 | 99.9 | y=1.014x+-114.6 | 99.9 | y=0.9765x+72.81 | 99.9 | y=1.226x+11.38 | 97.2 | y=0.8927x+43.31 | 85.8 | y=0.9991x+0.08496 | 90.7 |
| Parsnp | y=1.001x+-4.624 | 100.0 | y=0.9965x+1.43 | 100.0 | y=1.002x+-15.9 | 100.0 | y=0.9943x+7.423 | 99.9 | y=1.025x+-3.464 | 99.8 | y=0.9751x+-1.955 | 99.9 |
| kSNPCon | y=0.9993x+1.448 | 100.0 | y=0.9996x+-2.631 | 100.0 | y=1.001x+1.386 | 100.0 | y=0.9944x+3.687 | 99.8 | y=0.9856x+-0.05127 | 99.6 | y=1.002x+2.87 | 99.6 |
| kSNPRead | y=1.006x+5.66 | 99.9 | y=0.9476x+-8.046 | 100.0 | y=1.004x+-0.392 | 100.0 | y=1.006x+0.04707 | 100.0 | y=0.9825x+-0.1611 | 100.0 | y=1.003x+0.0208 | 100.0 |
| SMALTPpl | y=0.9965x+5.961 | 100.0 | y=0.9854x+-29.35 | 100.0 | y=0.9946x+14.68 | 100.0 | y=1.003x+2.033 | 100.0 | y=0.9949x+-3.413 | 100.0 | y=1.002x+4.114 | 100.0 |
| LyveSETRel | y=0.9298x+-8.165 | 99.9 | y=0.9267x+-28.02 | 99.8 | y=0.9319x+-10.97 | 99.9 | y=0.9433x+-1.266 | 99.2 | y=0.9044x+1.091 | 99.8 | y=0.8651x+2.399 | 99.5 |
| LyveSETStr | y=0.8763x+-15.55 | 99.7 | y=0.867x+-47.52 | 99.5 | y=0.893x+-18.71 | 99.9 | y=0.8849x+0.5828 | 99.8 | y=0.7693x+3.285 | 99.2 | y=0.8422x+2.034 | 99.8 |
| **Pipelines with recombination filtering** | | | | | | | | | | | | |
| ParsnpCl | y=0.9916x+-0.5866 | 100.0 | y=1.017x+-2.427 | 100.0 | y=1.002x+4.312 | 99.9 | y=1.023x+0.112 | 98.3 | y=0.7631x+3.548 | 97.9 | y=1.322x+-2.171 | 86.5 |
| ParsnpGu | y=0.9842x+-0.986 | 99.9 | y=0.8426x+4.746 | 99.1 | y=0.9681x+3.785 | 98.9 | y=0.6324x+4.301 | 60.5 | y=0.4257x+7.825 | 36.4 | y=0.7207x+5.212 | 48.8 |
| SMALTPplCl | y=0.9962x+-0.0823 | 100.0 | y=0.9854x+0.4355 | 100.0 | y=1.008x+-2.677 | 99.9 | y=1.007x+-0.2963 | 99.7 | y=0.9313x+0.3183 | 99.7 | y=1.077x+-0.07375 | 98.7 |
| SMALTPplGu | y=1.159x+-4.54 | 99.7 | y=1.359x+-14.13 | 97.4 | y=1.052x+2.329 | 99.5 | y=1.033x+0.5851 | 96.6 | y=0.7158x+1.04 | 97.8 | y=1.047x+0.1179 | 99.6 |

**Table S5. stability of the tested workflows towards inter-run variation of input sequencing data: phylogenetic tree topology.** The table displays topological distances between phylogenetic trees produced using replicate sequencing datasets. The analysis was carried out with the B:NT cc-269 isolate subsets from the two large datasets, B:NT cc-269 LDs **(A)**, the two small datasets, B:NT cc-269 SDs **(B)** and two composite small datasets, B:NT cc-269 cSDs **(C)**. The distances are calculated using the Kendall-Colijn (KC) test for topology (λ=0). Corresponding p-values, estimated by comparing the observed value to a distribution of KC distances of the query tree to 10^5^ random trees, are shown as -log(p). Bold: p<0.05.

|  | **A. Large datasets (LDs)** | | **B. Small datasets (SDs)** | | **C. Composite small datasets (cSDs)** | |
| --- | --- | --- | --- | --- | --- | --- |
| **Pipeline** | **KC (λ=0)** | **p** | **KC (λ=0)** | **p** | **KC (λ=0)** | **p** |
| cgMLST | 4.90 | -65.03 | 1.41 | -26.27 | 2.83 | -22.66 |
| PanseqBin | 86.06 | **-0.12** | 9.22 | -25.60 | 33.33 | **6.37201E-10** |
| PanseqSNP | 44.19 | -25.35 | 8.19 | -15.38 | 12.45 | -13.02 |
| Parsnp | 24.19 | -46.41 | 2.00 | -34.11 | 2.00 | -34.76 |
| kSNPCon | 30.53 | -35.61 | 4.36 | -25.77 | 3.46 | -30.58 |
| kSNPRead | 1.41 | -61.54 | 0.00 | -39.59 | 0.00 | -39.26 |
| SMALTPpl | 18.25 | -39.73 | 0.00 | -39.35 | 0.00 | -39.59 |
| LyveSETRel | 9.27 | -59.41 | 0.00 | -39.50 | 0.00 | -38.96 |
| LyveSETStr | 37.55 | -32.51 | 0.00 | -39.50 | 0.00 | -40.23 |
| **Pipelines with recombination filtering** | | | | | | |
| ParsnpCl | 40.93 | -28.99 | 3.32 | -23.67 | 3.32 | -27.10 |
| ParsnpGu | 84.36 | -22.70 | 13.34 | -9.58 | 5.10 | -26.02 |
| SMALTPplCl | 24.68 | -45.91 | 2.83 | -28.40 | 8.00 | -16.49 |
| SMALTPplGu | 39.03 | -37.40 | 5.10 | -20.67 | 0.00 | -35.15 |

**Table S6. Performance metrics of selected workflows with different reference genomes.** Performance metrics of selected workflows with three different reference genomes, more specifically Pacbio (/), hybrid Nanopore (np) and NC017515.1 (nc) were determined. The analysis was carried out with the B:NT cc-269 isolate subsets from the two large datasets, B:NT cc-269 LDs **(A)**, and the two small datasets, B:NT cc-269 SDs **(B)**. The table shows the discriminatory power (D), number of observed subtypes (subtypes), threshold used for the calculation of discriminatory power (TH), which corresponds to the maximal number of SNPs detected between replicate isolates, and the size of the genetic matrix (matrix size) calculated for the listed pipelines using different datasets.

|  | **A. B:NT cc-269 LDs** | | | | | **B. B:NT cc-269 SDs** | | | | |
| --- | --- | --- | --- | --- | --- | --- | --- | --- | --- | --- |
| **Pipeline** | **Data set** | **Matrix size** | **# sub types** | **D** | **TH** | **Data set** | **Matrix size** | **# sub types** | **D** | **TH** |
| Parsnp | LD1 | 33808 | 33 | 0.989 | 11 | SD1 | 17676 | 11 | 0.956 | 23 |
| Parsnp | LD2 | 33814 | 36 | 0.996 | 10 | SD2 | 17668 | 11 | 0.956 | 24 |
| Parsnp_nc | LD1 | 34120 | 34 | 0.990 | 6 | SD1 | 17997 | 11 | 0.956 | 17 |
| Parsnp_nc | LD2 | 34070 | 36 | 0.996 | 4 | SD2 | 18062 | 11 | 0.956 | 18 |
| Parsnp_np | LD1 | 33808 | 33 | 0.989 | 11 | SD1 | 17679 | 11 | 0.956 | 23 |
| Parsnp_np | LD2 | 33814 | 36 | 0.996 | 10 | SD2 | 17669 | 11 | 0.956 | 24 |
| SMALTPpl | LD1 | 34950 | 37 | 0.999 | 1 | SD1 | 18430 | 13 | 0.989 | 4 |
| SMALTPpl | LD2 | 34893 | 37 | 0.999 | 1 | SD2 | 18103 | 13 | 0.989 | 4 |
| SMALTPpl_nc | LD1 | 36128 | 37 | 0.999 | 0 | SD1 | 19634 | 13 | 0.989 | 4 |
| SMALTPpl_nc | LD2 | 36202 | 37 | 0.999 | 0 | SD2 | 19370 | 13 | 0.989 | 4 |
| SMALTPpl_np | LD1 | 35683 | 36 | 0.996 | 1 | SD1 | 18712 | 13 | 0.989 | 4 |
| SMALTPpl_np | LD2 | 35697 | 36 | 0.996 | 1 | SD2 | 18421 | 13 | 0.989 | 4 |
| **Pipelines with recombination filtering** | | | | | | | | | | |
| ParsnpCl | LD1 | 1669 | 37 | 0.999 | 1 | SD1 | 641 | 11 | 0.956 | 5 |
| ParsnpCl | LD2 | 1652 | 37 | 0.999 | 1 | SD2 | 642 | 13 | 0.989 | 5 |
| ParsnpCl_nc | LD1 | 1520 | 36 | 0.996 | 1 | SD1 | 586 | 12 | 0.967 | 5 |
| ParsnpCl_nc | LD2 | 1505 | 37 | 0.999 | 0 | SD2 | 594 | 13 | 0.989 | 5 |
| ParsnpCl_np | LD1 | 1672 | 37 | 0.999 | 1 | SD1 | 641 | 11 | 0.956 | 5 |
| ParsnpCl_np | LD2 | 1651 | 37 | 0.999 | 1 | SD2 | 642 | 13 | 0.989 | 5 |
| SMALTPplCl | LD1 | 1726 | 37 | 0.999 | 1 | SD1 | 657 | 14 | 1.000 | 0 |
| SMALTPplCl | LD2 | 1723 | 37 | 0.999 | 1 | SD2 | 650 | 14 | 1.000 | 0 |
| SMALTPplCl_nc | LD1 | 1571 | 37 | 0.999 | 0 | SD1 | 649 | 14 | 1.000 | 1 |
| SMALTPplCl_nc | LD2 | 1571 | 37 | 0.999 | 0 | SD2 | 623 | 14 | 1.000 | 1 |
| SMALTPplCl_np | LD1 | 1723 | 36 | 0.996 | 1 | SD1 | 670 | 14 | 1.000 | 0 |
| SMALTPplCl_np | LD2 | 1729 | 36 | 0.996 | 1 | SD2 | 664 | 14 | 1.000 | 0 |

**Table S7. Stability of selected workflows towards reference genome: Mantel test.** The table displays output of Mantel test accessing Spearman’s rank-order association (r^2^) and comparing pairwise SNP distances generated using replicate sequencing datasets. The workflows were tested with three different reference genomes, more specifically Pacbio (/), hybrid Nanopore (np) and NC017515.1 (nc). The analysis was performed for the B:NT cc-269, and the more closely related B:NT:P1.5,2* isolate subsets of two large datasets, LDs **(A)**, and two small datasets, SDs **(B)**.

|  | **B:NT cc-269**  **isolates** | | | **B:NT:P1.5,2* isolates** | | |
| --- | --- | --- | --- | --- | --- | --- |
|  | **A. LDs** | **B. SDs** | **A. LDs** | | **B. SDs** |  |
| **Pipeline** | **r^2^** | **r^2^** | **r^2^** | | **r^2^** |  |
| Parsnp | 100.0 | 99.4 | 97.1 | | 93.0 |  |
| Parsnp_nc | 100.0 | 99.3 | 97.2 | | 97.0 |  |
| Parsnp_np | 100.0 | 99.4 | 97.1 | | 93.0 |  |
| SMALTPpl | 100.0 | 99.5 | 98.7 | | 99.5 |  |
| SMALTPpl_nc | 100.0 | 99.8 | 99.4 | | 99.8 |  |
| SMALTPpl_np | 100.0 | 99.8 | 98.9 | | 98.6 |  |
| **Pipelines with recombination filtering** | | | | | | |
| ParsnpCl | 98.8 | 99.6 | 97.7 | | 99.3 |  |
| ParsnpCl_nc | 98.5 | 99.4 | 96.9 | | 94.3 |  |
| ParsnpCl_np | 98.8 | 99.6 | 97.7 | | 99.3 |  |
| SMALTPplCl | 99.8 | 99.6 | 99.9 | | 99.8 |  |
| SMALTPplCl_nc | 99.5 | 99.1 | 94.8 | | 92.4 |  |
| SMALTPplCl_np | 99.8 | 99.3 | 91.1 | | 97.8 |  |

**Table S8. stability of selected workflows towards reference genome: linear regression analysis.** The table displays output of linear regression analysis (equation, r^2^) comparing pairwise SNP distances generated by the different workflows using replicate sequencing datasets. The workflows were tested with three different reference genomes, more specifically Pacbio (/), hybrid Nanopore (np) and NC017515.1 (nc). The analysis was performed for the B:NT cc-269, and the more closely related B:NT:P1.5,2* isolate subsets of two large datasets, LDs **(A)**, and two small datasets, SDs **(B)**.

|  | **B:NT cc-269 isolates** | | | | **B:NT:P1.5,2* isolates** | | | |
| --- | --- | --- | --- | --- | --- | --- | --- | --- |
|  | **A. LDs** | | **B. SDs** | | **A. LDs** | | **B. SDs** | |
| **Pipeline** | **equation** | **r^2^** | **equation** | **r^2^** | **equation** | **r^2^** | **equation** | **r^2^** |
| Parsnp | y=1.001x+-4.624 | 100.0 | y=0.9965x+1.43 | 100.0 | y=0.9943x+7.423 | 99.9 | y=1.025x+-3.464 | 99.8 |
| Parsnp_nc | y=0.9982x+3.898 | 100.0 | y=0.9983x+13.14 | 100.0 | y=1.039x+4.153 | 99.9 | y=1.028x+-5.111 | 99.9 |
| Parsnp_np | y=1.001x+-4.625 | 100.0 | y=0.9965x+1.434 | 100.0 | y=0.9943x+7.423 | 99.9 | y=1.025x+-3.464 | 99.8 |
| SMALTPpl | y=0.9965x+5.961 | 100.0 | y=0.9854x+-29.35 | 100.0 | y=1.003x+2.033 | 100.0 | y=0.9949x+-3.413 | 100.0 |
| SMALTPpl_nc | y=1.001x+-6.239 | 100.0 | y=0.9888x+-31.24 | 100.0 | y=1.001x+-3.708 | 100.0 | y=0.9822x+-10.53 | 100.0 |
| SMALTPpl_np | y=0.9986x+-1.427 | 100.0 | y=0.9848x+-20.22 | 100.0 | y=1.006x+0.8118 | 100.0 | y=0.9932x+-1.465 | 100.0 |
| **Pipelines with recombination filtering** | | | | | | | | |
| ParsnpCl | y=0.9916x+-0.5866 | 100.0 | y=1.017x+-2.427 | 100.0 | y=1.023x+0.112 | 98.3 | y=0.7631x+3.548 | 97.9 |
| ParsnpCl_nc | y=0.988x+-0.9211 | 99.9 | y=1.067x+-3.806 | 99.8 | y=0.9774x+0.4595 | 96.8 | y=0.8742x+1.137 | 95.2 |
| ParsnpCl_np | y=0.9884x+-0.2666 | 99.9 | y=1.017x+-2.427 | 100.0 | y=1.023x+0.112 | 98.3 | y=0.7631x+3.548 | 97.9 |
| SMALTPplCl | y=0.9962x+-0.0823 | 100.0 | y=0.9854x+0.4355 | 100.0 | y=1.007x+-0.2963 | 99.7 | y=0.9313x+0.3183 | 99.7 |
| SMALTPplCl_nc | y=1.015x+-2.343 | 100.0 | y=0.9607x+-0.9803 | 99.9 | y=1.036x+0.2117 | 93.1 | y=0.9949x+-1.666 | 92.3 |
| SMALTPplCl_np | y=0.9959x+1.237 | 100.0 | y=0.9881x+-0.09194 | 100.0 | y=1.054x+-0.07336 | 96.3 | y=0.9968x+0.312 | 99.3 |

**Table S9. stability of the tested workflows towards reference genome: phylogenetic tree topology.** The table displays topological distances between phylogenetic trees produced using replicate sequencing datasets. The workflows are tested with three different reference genomes, more specifically Pacbio (/), hybrid Nanopore (np) and NC017515.1 (nc). The analysis is performed on the B:NT cc-269 isolates from two large datasets, B:NT cc-269 LDs **(A)**, and two small datasets, B:NT cc-269 SDs **(B)**. The distances are calculated using the Kendall-Colijn (KC) test for topology (λ=0). Corresponding p-values, estimated by comparing the observed value to a distribution of KC distances of the query tree to 10^5^ random trees, are shown as -log(p). Bold: p<0.05.

|  | **A. Large datasets (LDs)** | | **B. Small datasets (SDs)** | |
| --- | --- | --- | --- | --- |
| **Pipeline** | **KC (λ=0)** | **p** | **KC (λ=0)** | **p** |
| Parsnp | 24.19 | 47.44 | 2.00 | 34.39 |
| Parsnp_nc | 1.41 | 61.20 | 1.41 | 37.17 |
| Parsnp_np | 24.19 | 46.86 | 2.00 | 34.51 |
| SMALTPpl | 18.25 | 39.48 | 0.00 | 39.77 |
| SMALTPpl_nc | 2.45 | 63.14 | 18.19 | **0.35** |
| SMALTPpl_np | 2.00 | 63.10 | 0.00 | 40.04 |
| **Pipelines with recombination filtering** | | | | |
| ParsnpCl | 40.93 | 28.79 | 3.32 | 23.97 |
| ParsnpCl_nc | 5.74 | 62.40 | 0.00 | 36.63 |
| ParsnpCl_np | 40.90 | 28.97 | 3.32 | 23.95 |
| SMALTPplCl | 24.68 | 45.01 | 2.83 | 28.44 |
| SMALTPplCl_nc | 2.00 | 68.05 | 3.32 | 27.33 |
| SMALTPplCl_np | 27.20 | 41.55 | 0.00 | 36.06 |

# Bibliography

Darling, A. E., Mau, B., and Perna, N. T. (2010). progressiveMauve: Multiple Genome Alignment with Gene Gain, Loss and Rearrangement. *PLoS One* 5, e11147. Available at: https://doi.org/10.1371/journal.pone.0011147.

Okonechnikov, K., Conesa, A., and García-Alcalde, F. (2016). Qualimap 2: advanced multi-sample quality control for high-throughput sequencing data. *Bioinformatics* 32, 292–4. doi:10.1093/bioinformatics/btv566.

Ponstingl, H., and Ning, Z. (2010). SMALT-a new mapper for DNA sequencing reads. *F1000 Posters* 1, 313.
